# Bioinspired Additive Manufacturing of Hierarchical Materials: From Biostructures to Functions

**DOI:** 10.34133/research.0164

**Published:** 2023-06-09

**Authors:** Jingjiang Wei, Fei Pan, Hang Ping, Kun Yang, Yanqing Wang, Qingyuan Wang, Zhengyi Fu

**Affiliations:** ^1^ Institute for Advanced Materials Deformation and Damage from Multi-Scale, Institute for Advanced Study, Chengdu University, Chengdu 610106, P. R. China.; ^2^ Department of Chemistry, University of Basel, Basel 4058, Switzerland.; ^3^ State Key Laboratory of Advanced Technology for Materials Synthesis and Processing, Wuhan University of Technology, Wuhan 430070, P. R. China.; ^4^ College of Polymer Science and Engineering, Sichuan University, Chengdu 610065, P. R. China.

## Abstract

Throughout billions of years, biological systems have evolved sophisticated, multiscale hierarchical structures to adapt to changing environments. Biomaterials are synthesized under mild conditions through a bottom-up self-assembly process, utilizing substances from the surrounding environment, and meanwhile are regulated by genes and proteins. Additive manufacturing, which mimics this natural process, provides a promising approach to developing new materials with advantageous properties similar to natural biological materials. This review presents an overview of natural biomaterials, emphasizing their chemical and structural compositions at various scales, from the nanoscale to the macroscale, and the key mechanisms underlying their properties. Additionally, this review describes the designs, preparations, and applications of bioinspired multifunctional materials produced through additive manufacturing at different scales, including nano, micro, micro-macro, and macro levels. The review highlights the potential of bioinspired additive manufacturing to develop new functional materials and insights into future directions and prospects in this field. By summarizing the characteristics of natural biomaterials and their synthetic counterparts, this review inspires the development of new materials that can be utilized in various applications.

## Introduction

**Fig. 1.**
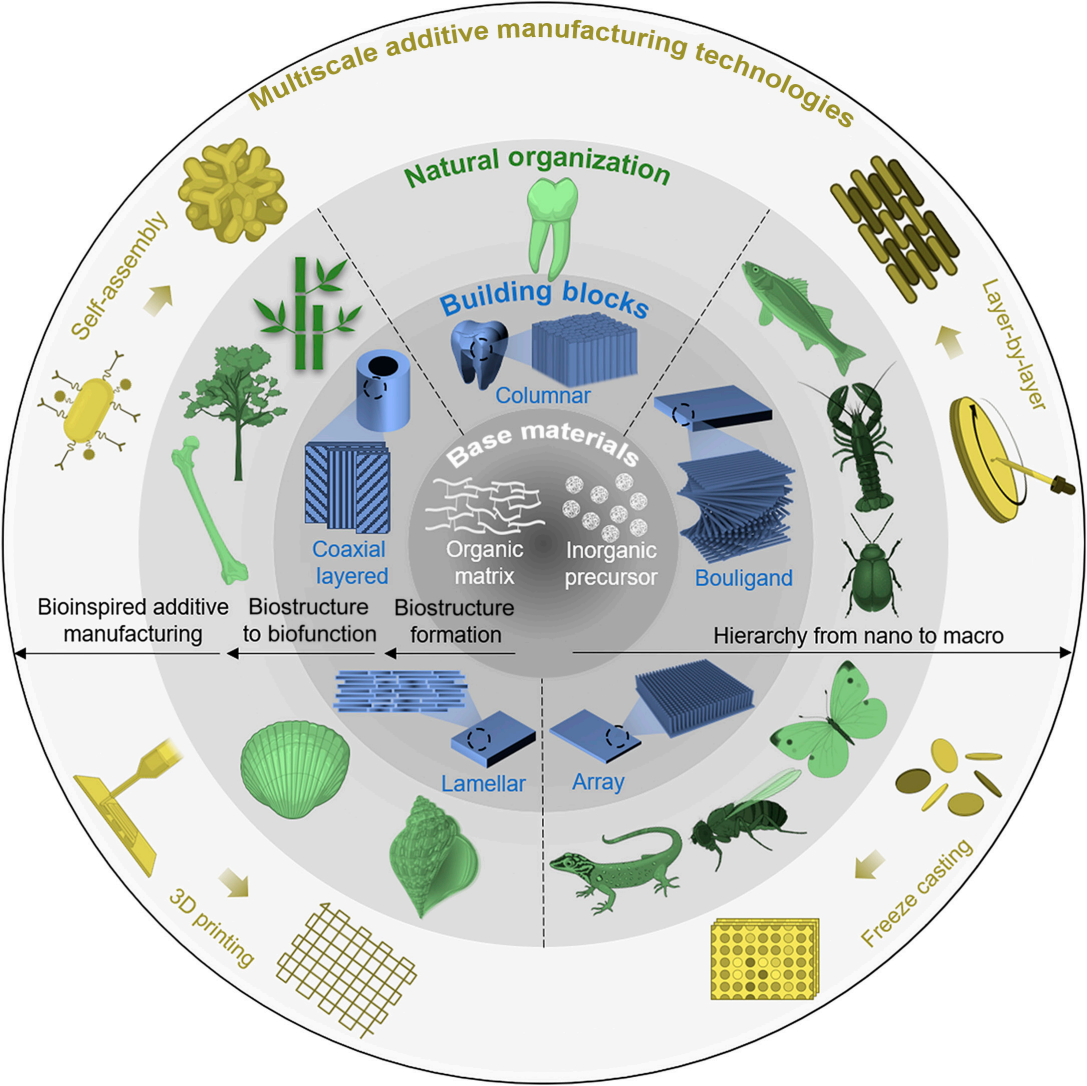
Multiscale additive manufacturing technologies. The building blocks of natural materials are primarily composed of an organic matrix and inorganic precursors. Various biomicrostructures result in the formation of different biofunctional materials. For example, (i) the coaxial layered structure leads to the formation of bone, wood, and bamboo; (ii) the columnar microstructure contributes to the formation of enamel; (iii) the Bouligand-type structure yields biomaterials such as fish scales, lobster claws, and insect shells; (iv) an array structure can be found in insect compound eyes; and (v) the lamellar arrangement exists in nacre and conch shells. These natural microstructures inspire the development of additive manufacturing technologies, including self-assembling, layer-by-layer, freeze-casting, 3D printing, and other related techniques.

Natural organisms face many challenges, including environmental changes, food acquisition, and predator threats [1–3]. Therefore, organisms have evolved biological materials with multifunctional properties beyond soft tissues [4]. Biomaterials are complex hierarchical structures composed of inorganic hard- and organic soft-ordered phases bridging the macroscale and the nanoscale [5–7]. All the multiscale structural models of the biomaterials evolved their innate functional features according to survival necessities [7–10]. For example, the lamellar arrangement found in nacre and conch shells endows them with outstanding mechanical strength and toughness (Fig. 1) [9,11–16]. The structure of enamel contains neatly arranged enamel pillars filled with the organic matrix. Such a structural design maintains striking similarities among numerous species (Fig. 1) [17–19]. Bone, wood, and bamboo have coaxial layered structures [1,5,8,20]. This structural arrangement exhibits not only excellent mechanical properties but also exceptional transport capabilities of matters (Fig. 1). Biological materials such as fish scales, lobster claws, and insect shells have a Bouligand-type structure (Fig. 1) [21]. Fish scales are a natural body armor with remarkable mechanical properties [22,23]. Lobster claws are a natural weapon with excellent impact resistance [24,25]. The Bouligand-type collagen fibers of insect shells exhibit outstanding mechanical properties and distinct structural color features [26–28]. Many species depend on physical characteristics besides mechanical ones to survive [29]. For example, from an optical perspective, changes in the microstructure of a chameleon’s skin can manipulate the color of the natural light it reflects, thereby achieving concealment [30,31]. The array structure of butterfly wings not only endows it with structural color but also exhibits excellent hydrophobicity [32–35]. Regarding visual sensory organs, the compound eyes of insects provide them with a full range of visual capabilities (Fig. 1) [36–38].

Learning from nature can discover new ways to solve technical bottlenecks in industry and life [39–46]. Natural biostructures are an origin of inspiration for designing next-generation structural materials with multifunctional properties [47–49]. The formation process of biological materials involves multiple physiological and biochemical processes of cells, fine regulation of genes and proteins, and bottom-up self-assembly using environmental resources [9,50]. This process occurs at ambient temperatures and pressures, making it economically and environmentally friendly. By gaining insight from the structure and formation process of biological materials, it is possible to design and develop new materials with high performance under room temperature and pressure [51].

However, the complexity of natural structures far surpasses conventional design and fabrication techniques, which poses a marked challenge for applying biomimetic materials in engineering systems [52,53]. Understanding the multilevel structure and formation process of biological materials reveals that organisms’ growth from small to large can be considered a natural additive manufacturing process directed by numerous multiscale biological processes that eventually yield the formation of a macroscopic shape conferring local heterogeneity [51,54–56]. Therefore, to prepare and develop biologically inspired materials, exploration and learning must be conducted from multiple scales, such as nano, micro, and macro [7]. However, designing and preparing structural materials with cross-scale properties using technical methods remains challenging in materials science and technology. Additive manufacturing, which includes self-assembling, layer-by-layer, freeze-casting, 3D printing, and other technologies, actively constructs structures, and its ability to build ordered microscale structures provides a feasible approach for developing new materials with bioinspired functions [52,53,57–59]. However, in the process of imitating the formation of natural biological structures and developing new materials with anticipated properties, cross-scale structures, and functions using additive technology, 3 critical scientific bottlenecks must be overcome: (a) understanding the mechanism of the formation process and multiscale structure of natural biological materials, (b) controlling bioinspired additive manufacturing processes in constructing cross-scale structures, and (c) understanding the connection between the structure and functional application of new materials developed by bioinspired additive manufacturing.

This review outlines current bioinspired multiscale additive manufacturing advancements for designing new multifunctional materials. The review is divided into 2 parts: (a) classification of typical biomaterial examples based on their microstructural characteristics, including lamellar arrangement, columnar alignment, coaxial layered arrangement, Bouligand structure, and array structure (their multiscale structures are summarized from molecular, nano, and micro to macro levels, alongside examples of the functionality endowed by these special biological structures), and (b) a summary of additive manufacturing technologies used to prepare biomimetic materials with targeted functions based on the classified multiscale structures of biomaterials. Finally, we discuss key mechanisms for developing new functional materials with cross-scale properties in multisystems through bioinspired additive manufacturing, current challenges, and prospects.

## Natural Hierarchical Structural Materials

It is well understood that the properties required for various material applications, such as those in the optical, mechanical, electrical, thermal, and magnetic fields, depend greatly on the composition and structure of the materials, which are directly related to the processing conditions and techniques [60–64]. Investigation into these properties facilitates the investigation of natural biomaterials’ structure, formation, and function [65].

**Fig. 2.**
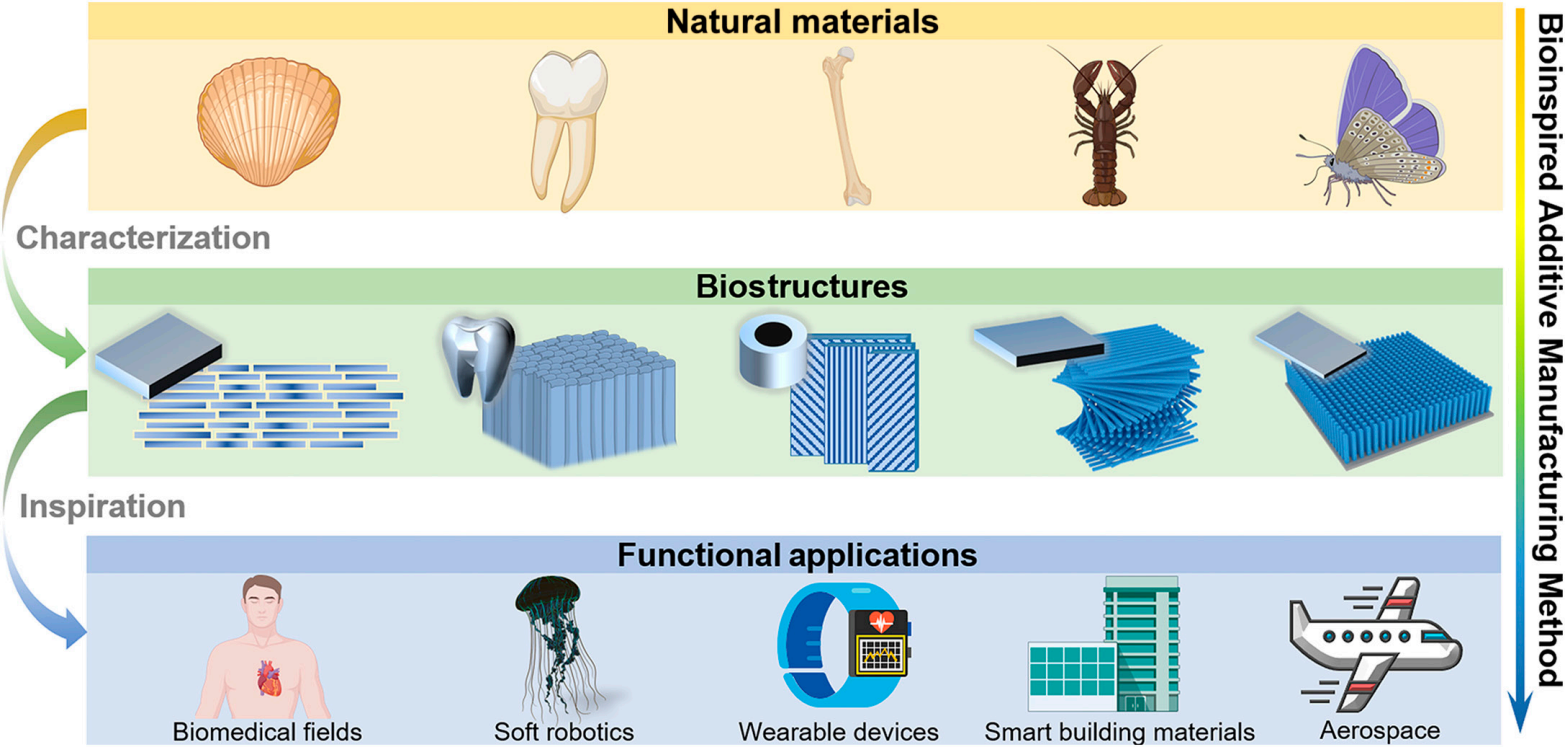
Bioinspired additive manufacturing techniques have emerged due to investigations into different biomicrostructures observed in natural materials such as shells, enamel, bone, and fish scales, including lamellar arrangement, columnar alignment, coaxial layered arrangement, Bouligand structure, and array structure. These techniques can potentially address challenges in various fields, such as artificial organs, soft robotics, wearable devices, smart building materials, and aerospace, by emulating the functionality of natural materials.

Organisms have utilized limited natural elements for billions of years to create various functional materials. These biomaterials display remarkable mechanical properties, as well as multiple functions, including self-healing and sensing [66–69]. Biomaterials typically feature composite structures that can be classified into soft and hard tissues. Soft tissues generally comprise soft biopolymers, such as proteins (including keratin, collagen, and elastin) and polysaccharides (e.g., hemicellulose, cellulose, lignin, and chitin) [70–77]. The hard tissue is usually further biomineralized on the natural organic framework to form a hard organic–inorganic composite material [5–7]. The multifunctionality of these natural composites can be attributed to their precise hierarchical assembly from bottom to top, resulting in complex and well-defined hierarchical structures at nano, micro, micro-macro, and macro scales [9]. In this section, we introduce the multiscale topologies of many typical biomaterials and summarize 5 representative structures and their respective application prospects. These structural elements can enhance mechanical properties and versatility (e.g., structural color, impact protection, and transmission), divided into lamellar arrangement, columnar alignment, coaxial layered arrangement, Bouligand structure, and array structure (Fig. 2). The lamellar arrangement can enhance the strength and toughness of natural ceramic-like tissues [78]. The columnar alignment is capable of protecting tooth tissue and reinforcing the anti-vibration performance and durability of the tissue [4]. The coaxial layered arrangement provides bending resistance and mass transport in mammalian bone, wood, and bamboo [5]. The Bouligand structure strengthens the toughness of animal shells [48]. The array structure contributes to structural color and hydrophobicity [7]. Inspired by the functionality of these biological structural materials, we expect to utilize additive manufacturing technologies to achieve breakthroughs in developing new functional biomimetic materials in various fields, such as artificial organs, soft robots, wearable devices, intelligent building materials, and aerospace (Fig. 2).

### Lamellar arrangement

The organized lamellar arrangement of biomaterials is characterized by an orderly lamella within biological tissues, established by the interplay of organic fiber frameworks and inorganic nanoparticles. This arrangement imparts biological tissues with exceptional strength, toughness, and wear resistance.

**Fig. 3.**
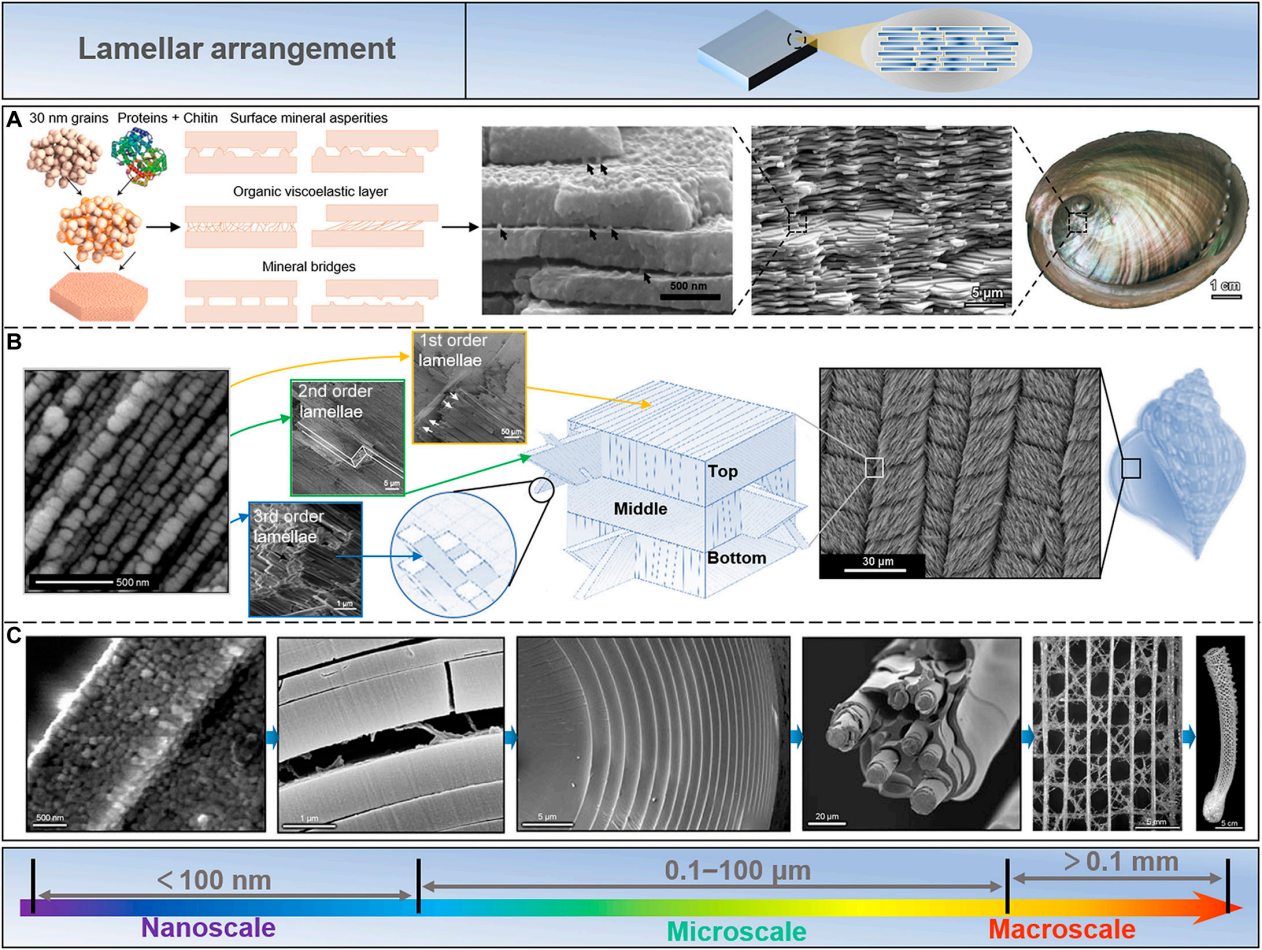
Lamellar arrangement in biomaterials. (A) Nacre, as a typical natural material, has a multiscale hierarchical structure. At the molecular level, the interaction of β-chitin and silk protein with 30-nm calcium carbonate particles facilitates the formation of a stable organic–inorganic structure. Reproduced with permission [5]. Copyright 2014, Springer Nature; Reproduced with permission [9]. Copyright 2008, Elsevier; Reproduced with permission [12]. Copyright 2008, Elsevier; Reproduced with permission [83]. Copyright 2011, Elsevier. (B) The conch shell evolves a 3-layer hierarchical lamellar structure to defend itself from the attack of predators. Each of these 3 layers manifests 4 distinct hierarchies at a nano-/microscale. Reproduced with permission [15]. Copyright 2017, Elsevier; Reproduced with permission [14]. Copyright 2013, Elsevier; Reproduced with permission [99]. Copyright 2017, Wiley VCH; Reproduced with permission [100]. Copyright 2003, Royal Society of Chemistry. (C) *Euplectella sponge* displays optical properties similar to artificial optical fibers while having unique strength and toughness. Moreover, this siliceous sponge confers multiple structural hierarchies from the nanoscale to the macroscale, contributing to its reinforced mechanical properties. Reproduced with permission [112]. Copyright 2005, American Association for the Advancement of Science; Reproduced with permission [113]. Copyright 2007, Elsevier.

Nacre, a composite material produced by certain mollusks as a protective shell against predators and environmental conditions, exhibits exceptional mechanical properties [9,79–81]. It is composed of mostly inorganic minerals (95 wt% aragonite) and a small amount of organic matrix (1 to 5 wt% *β*-chitin and silk fibroin) [11,82]. Nacre possesses a multiscale hierarchical structure (Fig. 3A) [5,12,47,83]. At the molecular level, a stable organic–inorganic structure is formed due to the interaction of *β*-chitin and silk protein with 30-nm calcium carbonate particles [84,85]. Many small calcium carbonate particles with the same crystal at the nanoscale constitute mesoscopic aragonite flakes. The aragonite flakes’ properties are enhanced due to the distribution of organic matter between the grains [86]. At the interface between the aragonite flakes (20 to 30 nm), nano-micro-protrusions on the surface reinforce the interfacial connections between the aragonite flakes, enabling them to withstand greater pressure or strain under bigger tension [87–89]. The sliding of aragonite flakes is limited by surface asperities of the flake nanominerals, the existence of mineral bridges, and the organic layer functioning as a viscoelastic binder [9,90–92]. At the microscale, nacre has a 3-dimensional (3D) “brick-and-mortar” structure, where the tightly connected polygonal aragonite flakes (5 to 8 μm in diameter and ~0.5 μm in thickness) are interconnected by organic materials with a thickness of 20 to 30 nm. The aragonite flakes filled and connected by organic layers between layers form a Thiessen polygonal pattern, conforming to the high amount of inorganic contents and the effectiveness of interfacial strengthening. Finally, in the continuous organic framework of chitin, the aragonite flakes are interconnected into a whole, forming the macroscopic nacre structure. The unique organic–inorganic multilevel hierarchical structure of nacre endows it with excellent mechanical properties. It has a fracture toughness that is roughly 3 orders of magnitude more than that of pure calcium carbonate mineral flakes because of its great tensile strength and Young’s modulus, which are in the ranges of 80 to 135 MPa and 60 to 70 GPa, respectively [93–95].

The conch shell forms a 3-layer hierarchical lamellar structure to resist the attack of predators, containing layered aragonite structures (99 vol%) combined with a biopolymer (1 vol%) [90,96–98]. The 4 separate hierarchies that comprise the inner, middle, and exterior layers have features ranging in scale from nanometer to millimeter (Fig. 3B) [14,15,99,100]. At the nanoscale, aragonite nanoparticles assemble single-crystal third-order sheets with sizes varying between 20 and 45 nm. These third-order sheets on organic frameworks exhibit ductility rather than brittleness. Third-order lamellae structures are the fundamental building blocks of conch shell formations at the microscale. These structures have cross-sectional diameters of 60 to 150 nm and 120 to 330 nm with a length of several hundred micrometers. The individual third-order sheets are combined with biopolymers to form second-order sheets with 5 to 30 μm thickness and 20 to 50 μm width. Similarly, second-order sheets stack together to form first-order sheets (10 to 70 μm in thickness and several micrometers in width). Stacking first-order lamellae structures horizontally constructs the conch shell’s microlamellar structures. The first-order lamellae’s orientations in adjacent microlamellar structures differ by 80° to 90°. This multiscale lamellar arrangement provides the conch shell with a 3D path for crack deflection and energy dissipation when it is impacted by external forces, resulting in high strength and toughness [97]. According to studies, the fracture toughness of the conch shell is 1,000 times greater than that of its mineral phase and 10 times greater than that of the nacre [97,101]. Because of these unique qualities, the conch shell is a fascinating target for biomimetic research into harder materials.

Glass is a widely used material known for its fragility; therefore, developing methods to strengthen glass has been an important area of research [102–108]. However, organisms have evolved effective ways of strengthening brittle materials, such as the silicon needles in sponges that exhibit excellent flexibility [109,110]. *Euplectella*, found in the western Pacific Ocean, possesses needles that exhibit not only optical properties comparable to artificial optical fibers but also unique strength and toughness [111]. This siliceous sponge contains multiple structural hierarchies from the nanoscale to the macroscale, all contributing to its enhanced mechanical properties (Fig. 3C) [109,112,113]. At the nanoscale, the primary building elements of the hierarchy are consolidated hydrated silica nanoparticles with a diameter of 50 to 200 nm that are connected but separated by protein filaments at the microscale. The layered structures allow the deep-sea sponge needles to stack concentrically, decreasing layer thicknesses from a center distance of 1.5 μm to a peripheral distance of 0.2 μm. Additionally, these silica needles (diameter, 5 to 50 μm) are arranged parallel to form bundles of needle aggregates. The aggregates of needles are cemented vertically and horizontally to form a square grid, yielding a centimeter-scale cylindrical cage-like structure of the deep-sea sponge.

In summary, the combination of ordered hierarchical arrangement and interfacial interactions in biomaterials is the main principle for designing the enhanced mechanical properties of natural materials. This delicate concept inspires construction of high-strength, high-toughness ceramic-like materials.

### Columnar alignment

**Fig. 4.**
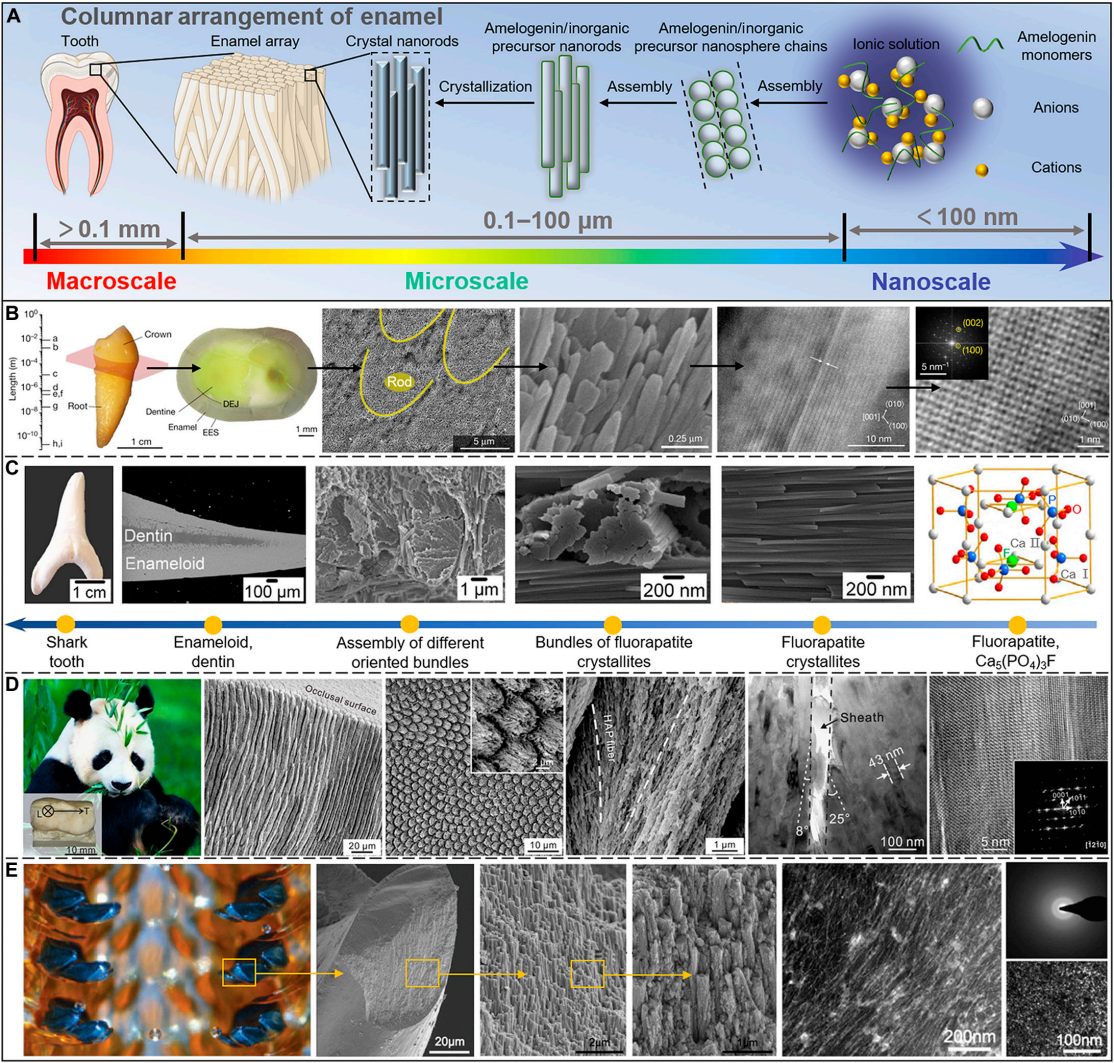
Columnar arrangement. (A) The tooth enamel is organized by enamel columns containing organic matrices. (B) The entire crown of human teeth covered by human tooth enamel manifests a thickness of several millimeters contributing to protecting the entire tooth tissue. Reproduced with permission [116]. Copyright 2020, Springer Nature. (C) The shark teeth display a hierarchical structure of 6 levels, while the organic matrix also displays a structural hierarchy of the mineral phases. Reproduced with permission [129]. Copyright 2014, Elsevier. (D) The teeth of a giant panda have exceptional resistance to damage because of the remarkable hierarchical structure toughening mechanism. Reproduced with permission [130]. Copyright 2016, Elsevier. (E) The teeth of *C. stelleri* have curved tricuspid structures. The inner teeth are comparatively soft hydrated ferric phosphate with a mineralized outer, consequently producing the highest hardness reported for biominerals. Reproduced with permission [139]. Copyright 2014, Wiley VCH; Reproduced with permission [137]. Copyright 2010, Elsevier.

The structure of tooth enamel displays neatly arranged enamel columns filled with organic matrices. Interestingly, this structure maintains striking similarities across species, for example, in human teeth, rat teeth, shark teeth, and even the enamel of walruses and dinosaurs [17,18,114]. Enamel’s intricate structure can be separated into numerous levels, ranging from the macroscale to the nanoscale [115,116]. As shown in Fig. 4A, at the nanoscale, the ionic liquid composed of amelogenin monomers, cations, and anions directionally self-assembles to form nanosphere chains of amelogenin/inorganic precursors. These nanosphere chains are further assembled into amelogenin-coated inorganic phase nanorods at the microscale. These nanorods crystallize to form hard nanorod crystal structures. The enamel layer is composed of an array of enamel rods arranged in an orderly manner and filled with an organic matrix. The enamel layer wraps the tooth’s outer surface at the macroscale, which endows the tooth with super durability and anti-vibration performance.

As shown in Fig. 4B, human tooth enamel covers the entire crown of human teeth, reaching a thickness of several millimeters, which helps to protect the entire tooth tissue [116,117]. The dentin–enamel junction (DEJ) enamel demonstrates a progressive transition from dentin to enamel. The prismatic regions of enamel are the original regions of the tooth because of their flexibility, which is considered the mechanism for their toughening [118,119]. Several investigators have identified these prismatic regions near the DEJ and on the apical surface of enamel in adult humans [120–122]. At the mesoscale, the main components of human tooth enamel are rod-like structures composed of well-aligned crystallites (~4 μm in diameter), whose crystallographic axes are oriented nearly parallel to the rods’ long axes. Interrod enamel, which envelops and surrounds nanorods, is the second structural element of human dental enamel, but its arrangement is less ordered. Such structures can withstand intensive impacts and resist damage from crack deflection. The third type of structure, prismatic enamel, comprises hydroxyapatite (HAP) crystals but lacks either mesoscale or macroscale ordering. In the presence of supersaturated ions, protein–protein and protein–mineral interactions produce a highly ordered array of HAP crystals that preferentially develop along the *c*-axis at the nanoscale. These HAP lattices have scales with typical lengths in the subnanometer region. Human dental enamel has a prominent mineral concentration (approximately 96 wt%), which contributes to its hardness (up to roughly 5 GPa) [123]. It has been reported to be more ductile than crystalline HAP, suggesting that the organization of microcrystals is crucial for human tooth enamel [124].

Compared with humans, who only change their teeth once in their lifetime, sharks will replace a row of teeth every 8 to 10 days and grow and replace about 20,000 teeth in their lifetime [125]. Moreover, unlike human enamel, shark enamel is composed of highly dense fluorapatite (FAP) crystals and a little amount of organic matrix (5 to 8 wt%), with a fluorine content of about 3.1 wt% [126]. Compared to HAP, FAP has a greater bulk modulus, stiffness constant, and elastic modulus, leading to shark teeth being harder than human teeth [127,128]. The structural hierarchy of shark teeth can be defined as 6 levels (Fig. 4C), and the organic matrix also follows the structural hierarchy of mineral phases [129]. The hexagonal FAP unit cell Ca_5_(PO_4_)_3_F is the smallest structural unit found in shark enamel. Ca_5_(PO_4_)_3_F forms hexagonal fluorapatite crystallites with a thickness of 50 to 80 nm and a length larger than 1 μm. When the crystallites are encapsulated by the organic matrix, they are tightly packed and arranged in bundles. The differently oriented bundles then form 3 distinct layers: the gloss layer, the enamel layer, and the intrinsic layer. Eventually, they form the whole macroscopic shark teeth with crown and root.

An endangered species, giant pandas eat virtually solely bamboo [130]. Bamboo is a natural material with a high fracture toughness and strength [131,132]. During the feeding process, the teeth of giant pandas exhibit remarkable damage resistance, which is attributed to their remarkable hierarchical structure toughening mechanism [130,133]. As shown in Fig. 4D, the length of the third premolar of the giant panda is 19 mm, the width is 11 mm, and the height is 6 mm. Its enamel consists of many micrometer-scale enamel rods, 5.4 ± 0.3 μm in diameter, which are internally oriented slant to form alternate intersecting bands, but are well aligned near the surface. The etched occlusal surface features a fish scale-like design, and individual rod cross-sections have a keyhole-type form. HAP nanofibers are arranged along the rod’s long axis in the middle and progressively slope toward the sheath at the rod’s perimeter. The deflection angle within the rod ranges from 8° on one side to 25° on the other. The interrod regions, or sheaths, are about 64 nm thick. Yet, because of the microscopic inhomogeneity of these sheath borders, certain fibers may extend from neighboring rods and collide there. According to high-resolution transmission electron microscopy (HR-TEM) and fast Fourier transform patterns of the matching isolated single crystals, the fibers have a hexagonal apatite phase structure. The fibers’ long axis and the HAP [0001] crystal orientation match quite well. Such a hierarchical structure makes the enamel cracks always propagate along the internal interface when impacted, especially the sheaths between the enamel rods. Such shields the fracture from applied stress, enhancing damage resistance and external toughening mechanisms, including crack deflection/torsion and crack-free ligament bridging.

One of the animal kingdom’s most wear-resistant structures comes from the sea snail known as the chiton [134,135]. Their teeth are attached to the organic membrane and arranged in parallel to form a radula [136]. The tooth of a chiton is a complicated, layered arrangement of biological complexes with a sophisticated design encompassing several length scales [137–139]. The teeth of *Cryptochiton stelleri* are composed of tricuspid curved structures at the macroscale (Fig. 4E). When chitons eat, their teeth repeatedly scrape the algae on the rock surface, requiring excellent wear resistance [135]. Thus, the inside of the teeth of *C. stelleri* is relatively soft hydrated ferric phosphate, and the outside is mineralized with a crust of magnetite, showing the highest hardness reported for biominerals [136–138,140,141]. A cross-section of a chiton tooth reveals how magnetite crystallites are organized. Scanning electron microscopy (SEM) revealed that the magnetite was organized into grain bundles approximately 250 nm wide, parallel to the tooth’s long axis, and all surrounded by a thin organic layer. Moreover, the TEM investigation supported the core material’s weakly crystalline and organic-rich structure as well as the magnetite crystallites’ rod-like orientation.

In addition to the enamel, the prismatic layer of the shell is also a natural columnar structure, mainly composed of calcite [81]. Compared with the nacre layer, the prismatic layer is relatively less studied. Similar to the nacre layer, although a small amount of inorganic phase is sacrificed, adding a small amount of organic matter enormously improves its hardness, almost 1.5 times that of pure calcite [142].

The columnar structure of tooth enamel has evolved in nature and likely has a important purpose. Understanding its formation process may enable the development of materials with analogous structures and functions, with potential applications in biomedical engineering and aerospace.

### Coaxial layered arrangement

Bone, wood, bamboo, and sheep horn exhibit good mechanical strength and material transport capacity because they have a natural multilevel coaxial multilayer structure with different fiber orientations, namely, coaxial layered arrangement.

**Fig. 5.**
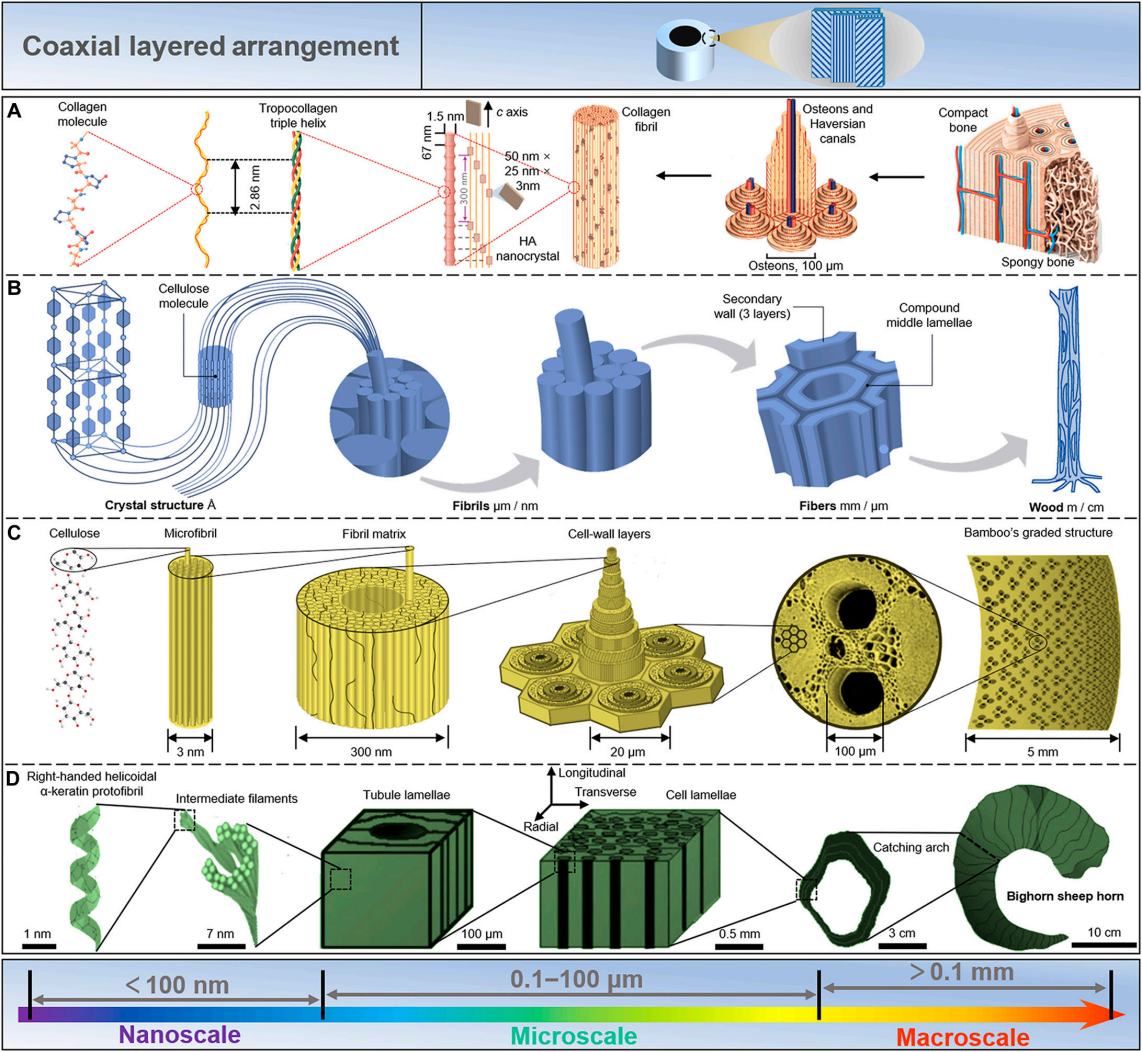
Coaxial layered arrangement. (A) Bone is a hierarchical structural material with mineralized collagen fibers as the basic building blocks. Reproduced with permission [5]. Copyright 2014, Springer Nature. (B) Wood and bamboo display a coaxial layered arrangement. In the different layers of the cell wall of wood fibers, each fibril exhibits a chain of crystalline units linked together by amorphous domains. Reproduced with permission [146]. Copyright 2004, Wiley VCH. (C) Bamboo can further optimize the fiber density distribution based on the coaxial layered structure reaching a higher mechanical efficiency against bending. Reproduced with permission [5]. Copyright 2014, Springer Nature. (D) The horn of *O. canadensis* has a basic building composed of *α*-keratin, a fibrous structural protein, which can be further assembled into intermediate filaments and embedded in a protein matrix, thereby forming sheets. Reproduced with permission [153]. Copyright 2010, Elsevier.

Bone is a highly vascularized, dynamic tissue made up of 70% mineral (mainly nanoscale HAP crystals) and 30% organic matter (including collagen, glycoproteins, proteoglycans, and salivary proteins) [143]. It is a lightweight, high-strength, high-toughness, self-healing natural composite material. As shown in Fig. 5A, bone is considered a hierarchical structural material with mineralized collagen fibers as the basic building blocks [5]. The first layer is a fibrous collagen formed by collagen molecules of type I (300 nm in length and 1.5 nm in diameter). The second layer is a fiber bundle with a helical structure formed by 3 bundles of fibrous collagen molecules, with certain gaps in between. At the third level, calcium phosphate begins to grow confinement from the gaps, and these growing HAP nanocrystals (plate-shaped, 50 nm × 25 nm × 3 nm) orientate in a preferred direction, maintaining the *c*-axis parallel to the collagen fibers. At this stage, megapascals of prestress are created within the collagen fibers [144]. At the fourth level, HAP nanocrystals gradually wrap the entire fiber bundle to form mineralized collagen fiber bundles. These fibers are rarely found in isolation but always in bundles or other connected arrangements along the long axis. Then, at the fifth level, these mineralized collagen fibers are organized into hollow structures called Havers’ canals, which serve as nerve and blood supply channels for bone cells. The macroscale division of bone tissue into spongy (cancellous bone) and dense (cortical bone) is the sixth level of bone tissue. Cancellous bone has a distinctly lower compressive strength but is very porous (75 to 95% porosity), allowing room for bone marrow and blood vessels. A thick outer layer (5 to 10% porosity) of cortical bone performs supporting functions [145].

Both wood and bamboo are composed of coaxial layered arrangements. As shown in Fig. 5B, in the different layers of the cell wall of wood fibers, each fibril can be considered a chain of crystalline units linked together by amorphous domains [146–148]. These high-tensile-strength cellulose molecules are embedded in the matrix substance lignin to form microfibrils. The diameter of the fiber is at the nanoscale, with the length at the microscale. The microfibrils are arranged into compact assemblies due to interchain and intrachain hydrogen bonds, which are facilitated by the abundant hydroxyl groups of cellulose. Hence, the yielded structures span different dimensions and levels. To obtain improved mechanical efficiency against bending, bamboo further refined the fiber density distribution based on the coaxial layered structure (Fig. 5C) [5,20,149,150].

The horns of *Ovis canadensis* have a similar structure and are subjected to high-impact loads when combating other animals [151]. However, they must absorb impact energy to minimize its transfer to the animal’s bones. This high-impact resistance of a horn is derived from its hierarchical structure at different length scales [152]. As shown in Fig. 5D, the basic building block of the horn of *O. canadensis* is *α*-keratin, a fibrous structural protein [153]. They are further assembled into intermediate filaments and embedded in a protein matrix, thereby forming sheets [154]. Long thin tubes are interspersed between the sheets along the length of the corners. The final structure is a 3D laminated composite of fibrous keratin with a porosity gradient running the length of the horn.

In summary, the mechanical strength and material transport ability exhibited by the coaxial layered configuration may be used in crucial ways in architecture and mechanical engineering.

### Bouligand structure

The Bouligand structure comprises continuous unidirectional nanofiber sheets helically stacked, displaying a certain rotation angle between adjacent fiber layers, a typical fiber-reinforced structure. This structure is widely found in various biological materials such as fish scales, insect shells, and lobster claws.

**Fig. 6.**
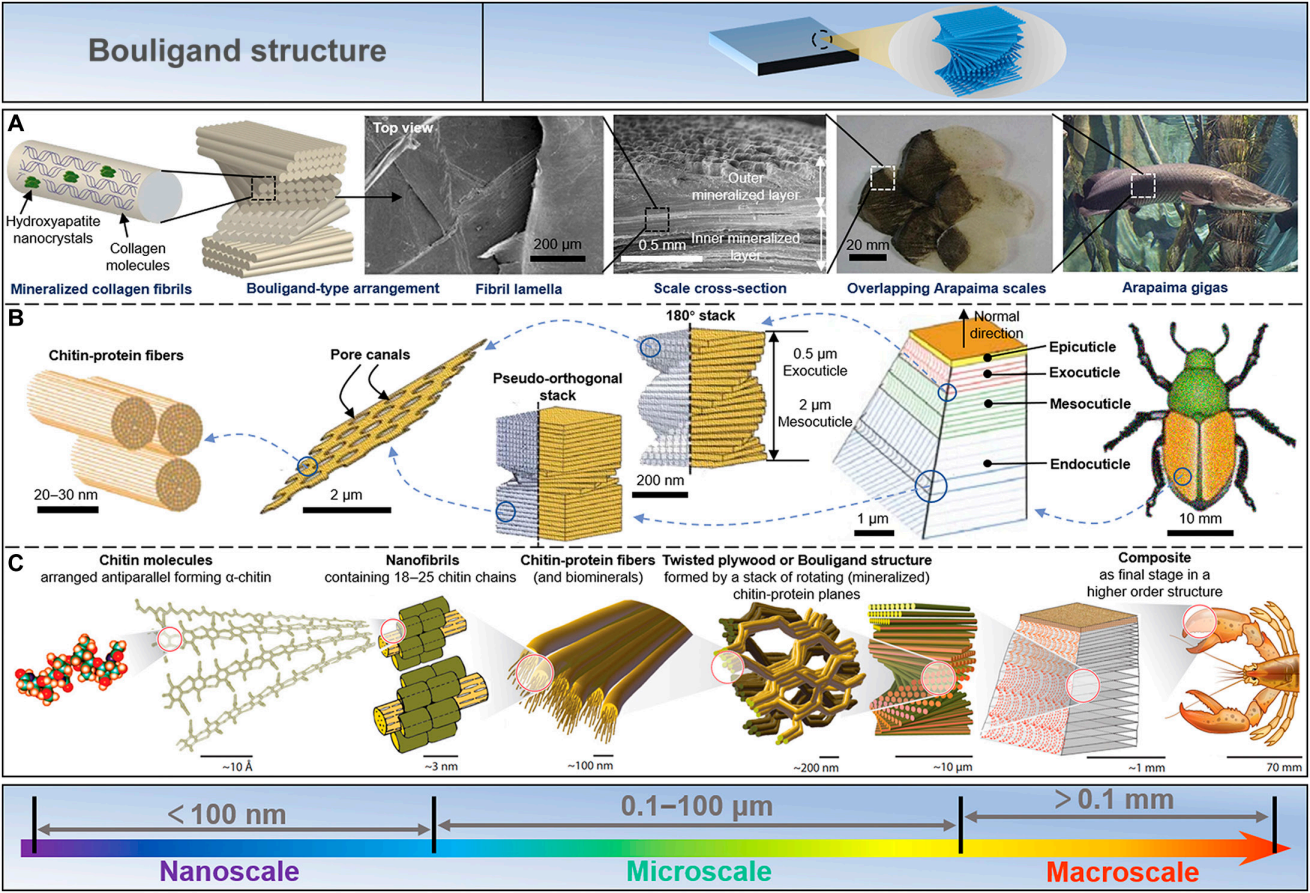
Bouligand structure. (A) The natural hierarchical Bouligand structure of *Arapaima ginas*’ scales yields the strength and toughness to defend itself from predation. Reproduced with permission [155]. Copyright 2013, Elsevier. (B) In investigating an insect (*Japanese beetle*), its exoskeleton consists of 4 regions: epicuticle, exocuticle, mesocuticle, and endocuticle. Reproduced with permission [163]. Copyright 2009, Springer Nature. (C) The lobster claws can be divided into 5 levels, from the nanoscale to the macroscale. The nanofibers in lobster claws contain α-chitin chains composed of N-acetylglucosamine molecules. Subsequently, the proteins bind to chitin nanofibers and self-assemble into Bouligand structures, layers of mineralized fibers stacked by helices at an angle. Reproduced with permission [164]. Copyright 2005, Elsevier.

*Arapaima gigas* is protected against piranhas by the hardness and strength of its scales, which function as natural leather armor, owing to its scales’ natural hierarchical Bouligand structure (Fig. 6A) [22,23,155]. At the macroscale, the scales’ aspect ratio (length/thickness) and degree of overlap determine that they can bend freely, providing them the flexibility to move [156]. Mesoscopic scales have scaled material qualities to resist bending or puncturing across their thickness [157–159]. It is divided into 2 separate regions: an inner core area made of collagen and a highly mineralized outer shell around 0.5 mm thick (approximately 1 mm thick) [160,161]. At the microscale, the interior is lined with multiple layers of approximately 50-μm-thick collagen sheets assembled from mineralized collagen fibrils. The orientation of fibrils within each lamella is consistent but misaligned in adjacent lamellae, forming a so-called Bouligand structure [160–162]. This Bouligand structure enhances the ductility and toughness of the scales to avoid fracture by allowing the flakes to reorient in response to the loading environment.

Insects in armor look as bulky as turtles. In fact, the hard shells that protect the wings are so light that they fly effortlessly, thanks to the Bouligand structure of the shell of insects. As shown in Fig. 6B, by studying a common insect (*Japanese beetle*), it was found that its exoskeleton consists of 4 regions, including epicuticle, exocuticle, mesocuticle, and endocuticle [163]. The porous chitin fiber sheets of the exocuticle and mesocuticle are arranged in a helical structure with a pitch of about 0.5 μm and 2 μm, respectively. The endocuticle has a characteristic pseudo-orthogonal structure defined by a narrow transitional helical area sandwiched between 2 orthogonally stacked layers. These 3 regions are primarily responsible for weighing and consist of layers of oriented chitin-protein fibers parallel to the surface of the stratum corneum. Such material composition and structure thus exhibit the properties of lightweight and high strength.

In addition to being used as a protective “shield” for animals, the Bouligand structure can also be utilized to assault the target as a “spear.” The *Homarus americanus* (American lobster) is a large crustacean that uses its claws to attack and grab food. From the nanoscale to the macroscale, lobster claws can be divided into 5 levels (Fig. 6C) [24,25,47,164–166]. N-acetylglucosamine molecules are aligned antiparallel at the nanoscale to *α*-chitin chains. Subsequently, 18 to 25 chitin chains self-assemble to form nanofibers. At the microscale, these proteins bind to chitin nanofibers and subsequently self-assemble into Bouligand structures, which are layers of mineralized fibers stacked by helices at an angle. These mineralized fiber layers are further stacked at the macroscale to form a biocomposite, which displays excellent impact resistance.

Bouligand structures exist in different species, but all manifest an enhanced toughness through interlayer coupling, efficient stress transfer, and twist crack propagation. A thoughtful and in-depth understanding of natural materials’ Bouligand structure and properties undoubtedly helps us design new, lighter, and stronger materials.

### Array structure

In addition to the aforementioned biomaterials, some natural biomaterials display unique functions, such as structural color, visual ability, and hydrophobicity. The natural array structure endows these biomaterials with special functions.

**Fig. 7.**
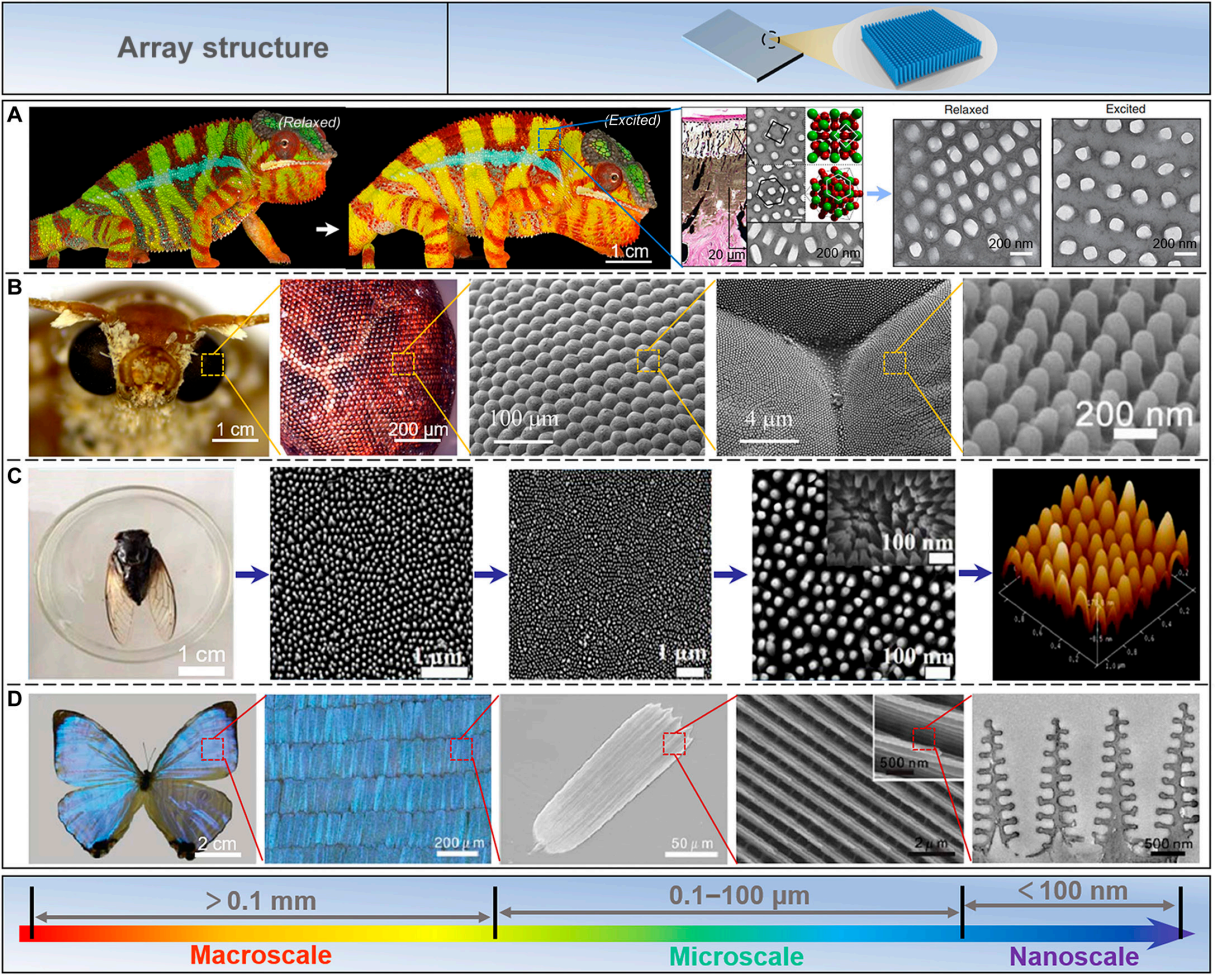
Array structure. (A) Chameleons are stimulated to change the undertone of their skin from green to yellow or orange. Reproduced with permission [31]. Copyright 2015, Springer Nature. (B) The compound eyes of moths yield a supernatural macroscopic function caused by their special microstructure and morphology. Reproduced with permission [177]. Copyright 2022, Springer Nature; Reproduced with permission [38]. Copyright 2016, American Chemical Society. (C) The microscopic structures on the surface of the cicada wing manifest nanopapillary arrays. Reproduced with permission [185]. Copyright 2017, AIP Publishing. (D) The butterfly (*M. sulkowskyi*) wings display a bright blue iridescence from a complex, layered photonic structure. Reproduced with permission [188]. Copyright 2018, Royal Society of Chemistry.

*Furcifer pardalis*, a particular chameleon species, is known for its intricate and quick skin color changes [30,31]. These changes were thought to be caused by the dispersion/aggregation of pigment-containing organelles within the dermal chromophore [167,168]. In fact, chameleons change color by actively adjusting the lattice alignment of guanine nanocrystals in the thick superficial layer of their dermal iris carrier [30,31]. As shown in Fig. 7A, when motivated, adult male panther chameleons may actively alter their background skin color from green to yellow or orange [31]. This procedure takes place quickly and is completely reversible. According to histological and TEM studies, the skin is composed of 2 overlapping layers of iridescent cells holding guanine crystals of various sizes, shapes, and structures. Only mature male skin has a completely formed top layer with densely packed guanine crystals (127.4 ± 17.8 nm in diameter) [169]. This arrangement of materials of high and low refractive indices allows chameleons to easily change the color of their skin.

Moths can see objects clearly in the dark, attributed to their special compound eye structure [38,170,171]. To prevent abrupt changes in the refractive index of incoming light, these complex eye structures serve as an efficient continuous gradient between the air and the ocular medium [172]. As shown in Fig. 7B, further studies have shown that the supernatural macroscopic functions exhibited by the compound eyes of moths are derived from their special microstructure and morphology [173–177]. The natural eye of moths is a subwavelength structure with 250-nm high protrusions (called corneal papillae) with a period of 300 nm and is arranged in a highly ordered hexagon [178]. One of nature’s most potent antireflective coatings, the moth-eye pattern is made possible by the structural makeup of such multiscale nanoarrays.

Similarly, the surface of cicada wings is also a highly ordered array of nano-nipples, which raises scientists’ great interest [179–182]. This structure minimizes light reflection over a broad wavelength range, thereby increasing photon collection and reducing reflectivity [183]. In addition to this high-performance optical property, the flap also possesses well-known superhydrophobic and self-cleaning properties [184]. Microscopically, the average spacing of the nanopapillary arrays on the surface of cicada wings was 160 nm, the height was 200 nm, the top was 60 nm, and the bottom diameter was 140 nm (Fig. 7C) [185]. Chitin makes up the majority of cicada wings (7 to 9 GPa) [186].

Structural colors in nature are visually pleasing, caused by the constructive and destructive interference of light when the light interacts with ordered arrays of nanoscale high-refractive-index materials such as keratin, chitin, or cellulose [32,33,187]. The butterfly (*Morpho sulkowskyi*) wings in Fig. 7D display a bright blue iridescence stemming from a complex, layered photonic structure [188]. A scale is the basic unit of color on a butterfly’s wing, with a grid of ridges and transverse ribs. The scales are composed of chitin, about 200 μm long and 50 μm wide. TEM images reveal details of the scales having Christmas tree-like structures. Each nanostructure consists of a backbone with a width of 50 to 120 nm and many small lamellae of 65 nm.

Collectively, the natural array structure is a great inspiration for us to develop advanced multifunctional materials.

## Multiscale Additive Manufacturing Technologies of Biomimetic Materials

**Fig. 8.**
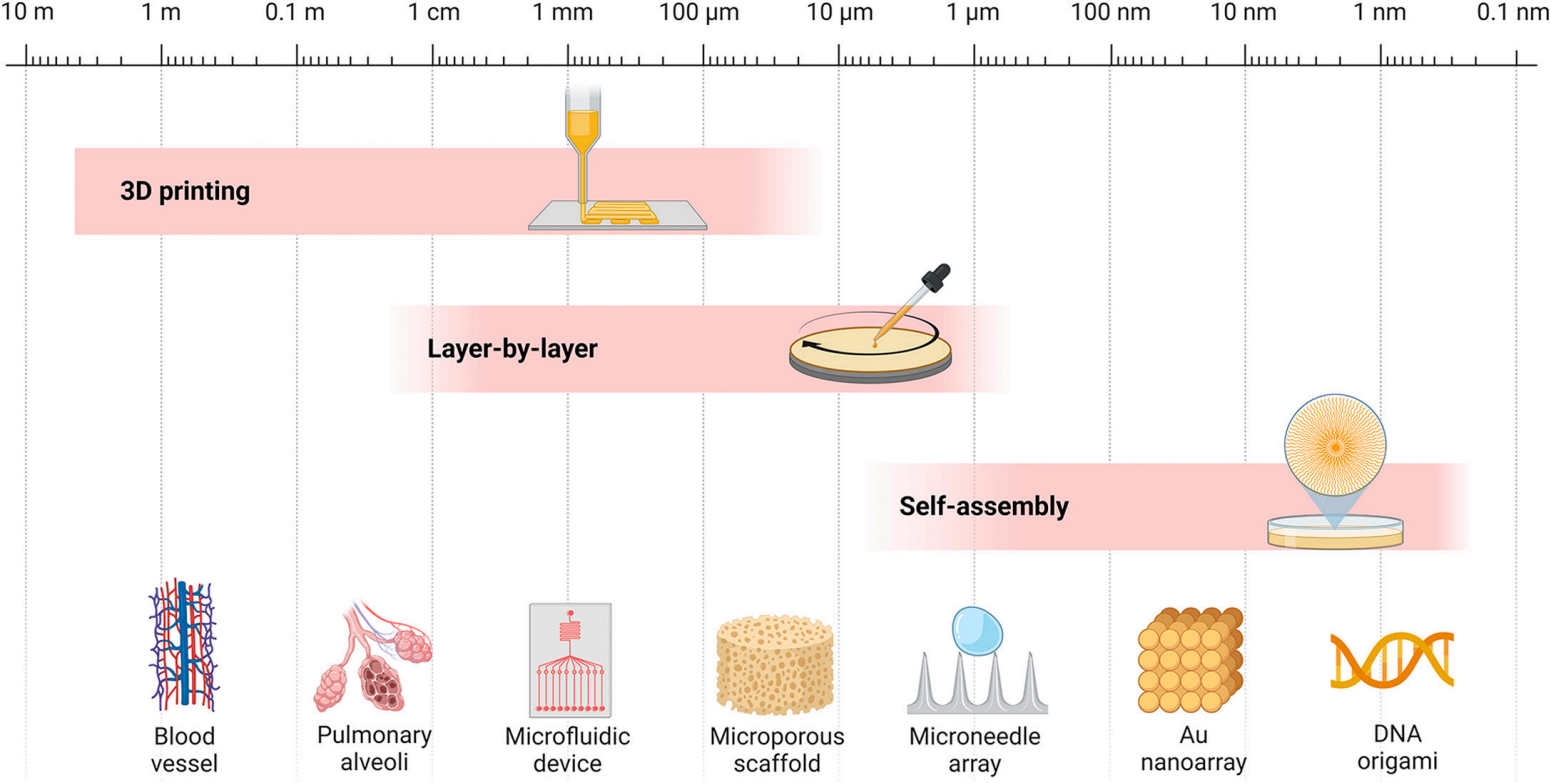
Various additive manufacturing technologies have been developed for different applications, including self-assembling, layer-by-layer, and 3D printing. These techniques have enabled the fabrication of various complex structures such as artificial blood vessels, pulmonary alveoli, advanced microfluidic devices, microporous scaffolds, microneedle arrays, Au nanoarrays, and DNA origami.

The general strategy for developing biologically inspired materials involves selecting a particular biological material’s unique function or structure, investigating it thoroughly, and establishing the relationship between structure and functionality [4,5,10,47,48,189]. Based on the acquired knowledge, a suitable material system and preparation method can be selected to replicate the biological structure and obtain similar functions or comparable properties to biological materials [56,190–193]. However, biomaterial formation entails multiscale processes from the nanoscale to the macroscale. For instance, the biomineralization process of shell growth progresses from small to large scale, resulting in a final product with an almost coherent appearance. A similar biomineralization growth process also shapes teeth and bones. Nature has used additive manufacturing evolutionary methods for billions of years to create high-precision and refinement hierarchies to meet specific needs [194,195]. This natural additive manufacturing process, guided by biological processes, generates macroscale forms with regional variability and produces refined structures far superior to their laboratory-developed counterparts. As illustrated in Fig. 8, researchers have made remarkable strides in fabricating biomimetic composites using various additive manufacturing methods and techniques, including self-assembly, layer-by-layer, 3D printing, and freeze-casting [8,196–198]. However, additive manufacturing techniques at all scales have their advantages and disadvantages. Nano-additive manufacturing technology can accurately construct nanoscale lattice structures, but it is often difficult to scale production. Micro-additive manufacturing technology can be used to fabricate microstructures controllably, but currently, available materials are limited and cannot support a wide variety of materials. Macro-additive manufacturing methods can scale up complex shapes, but the current applications of printed materials are limited. Hence, the reasonable combination of multiscale additive manufacturing technology and bioinspired preparation principle may be a path to develop new functional materials. Therefore, this section explores the research status and application of biomimetic additive manufacturing technology in the nano, micro, micro-macro, and macro scales.

### Nanoscale additive manufacturing

Deoxyribonucleic acid (DNA), an essential biomacromolecule for living organisms, carries biological and genetic information [199]. It is a lengthy polymer comprising nucleotide units with a length of 0.33 nm and a chain width of 2.2 to 2.6 nm [200]. In the molecular structure of DNA, 2 polydeoxynucleotides are wound around a common central axis, forming a double-helix structure. The 2 polydeoxynucleotide strands are reverse complementary and joined by base pairing formed by interbase hydrogen bonds, forming a fairly stable combination [201].

**Fig. 9.**
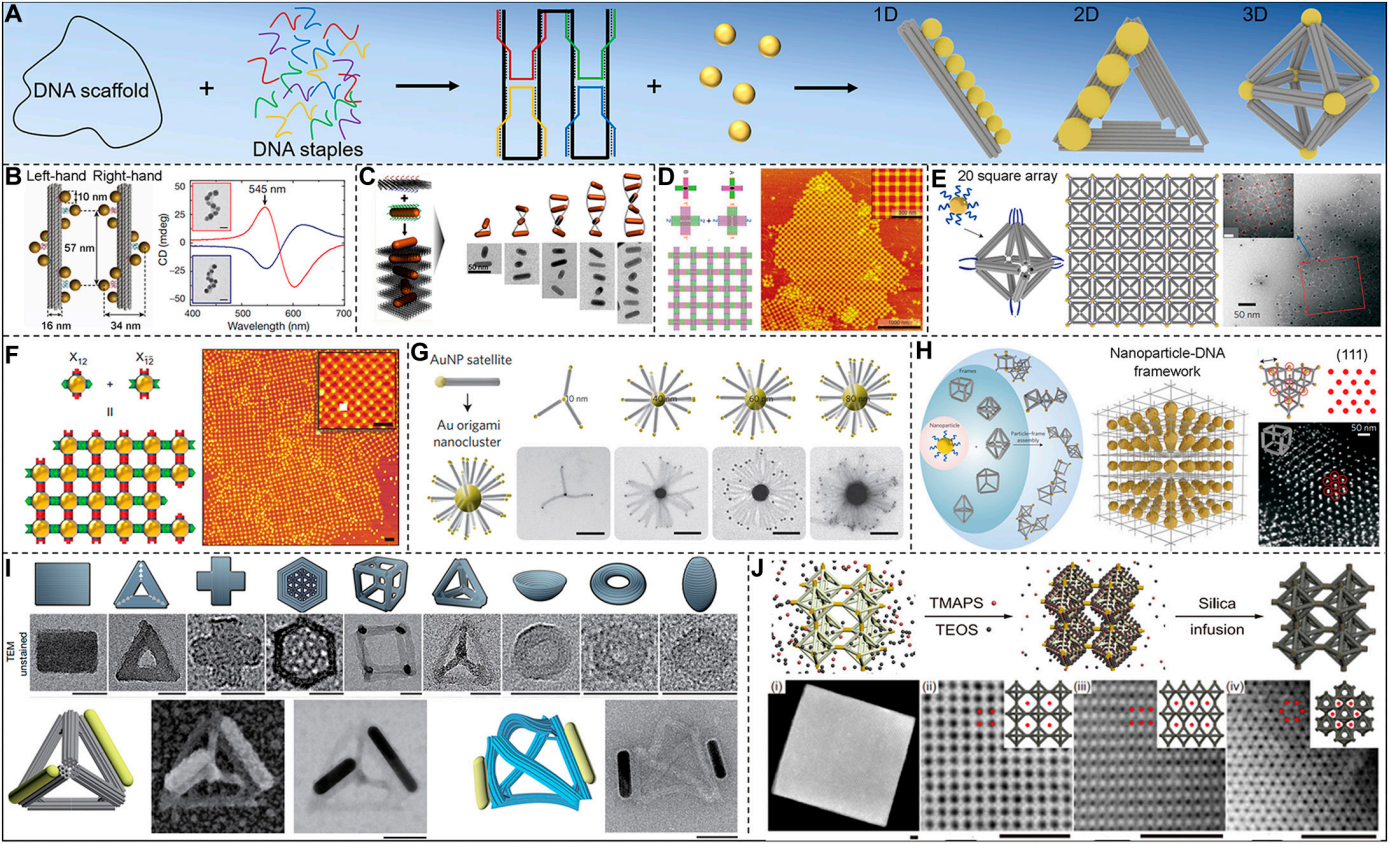
Overview of DNA origami-based nanoscale additive manufacturing design strategies and structures. (A) Overview of DNA origami-based nanoscale additive manufacturing design strategies and structures. (B) Assembled chiral AuNP helix interactions with polarized light on DNA origami templates. Reproduced with permission [205]. Copyright 2012, Springer Nature. (C) Self-assembled chiral AuNR. Reproduced with permission [210]. Copyright 2015, American Chemical Society. (D) 2D AuNP arrays assembled by DNA origami. Reproduced with permission [211]. Copyright 2010, Wiley VCH. (E) 2D AuNP square arrays. Reproduced with permission [214]. Copyright 2015, Springer Nature. (F) 2D AuNP arrays assembled DNA origami frames. Reproduced with permission [218]. Copyright 2016, Springer Nature. (G) Planet–satellite Au nanocluster assembled by DNA origami. Reproduced with permission [221]. Copyright 2013, Springer Nature. (H) Au superlattices assemble through DNA frameworks. Reproduced with permission [222]. Copyright 2016, Springer Nature. (I) DNA origami silicification nanostructure with a pattern similar to the cell wall unit of diatoms. Reproduced with permission [226]. Copyright 2018, Springer Nature. (J) Silica-encapsulation strategy for free-standing DNA cubic microcrystal. Reproduced with permission [227]. Copyright 2021, Springer Nature.

In fact, the DNA formation process is a typical natural nanoscale additive manufacturing process [202,203]. One technique of particular relevance to nano-additive manufacturing is the method of DNA origami. In 2006, Rothemund [204] took advantage of the uniqueness and predictability of Watson–Crick base pairing, used hundreds of DNA single strands on a DNA scaffold chain for complementary pairing, and thus constructed almost arbitrary 2D/3D shapes on the nanometer scale. The self-assembling nanofabrication method based on DNA origami enables the mass development of 1D, 2D, and 3D nanoscale devices at a reasonable cost (Fig. 9A).

DNA origami technology confers the efficient organization of ordered arrangements of gold nanoparticles (AuNPs) and thus manipulates light. Liedl and colleagues [205] used 24-helix bundles of DNA origami to self-assemble 9 AuNPs of around 10 nm into right-handed or left-handed helices (Fig. 9B). Such structures display switchable circular dichroism and optical dispersion effects at visible wavelengths, with localization precision higher than 2 nm, resulting from the collective plasmon–plasmon interactions of the nanoparticles. Similarly, Ding et al. [206] showed how to create 3D plasmonic chiral nanostructures via programmable switching of DNA origami modified with AuNP. Liu and colleagues [207] constructed plasmonic toroidal supramolecules with a tunable optical activity using 4 identical helical construction pieces inspired by an origami design. This toroidal supramolecule shows a stronger chiral optical response than the monomers and dimers of the helical building block. Their work provides a new strategy for building plasmonic chiral platforms with complex nanostructures. AuNPs and nanorods exhibit intriguing optical characteristics that can be changed by interaction [208,209]. To address the challenge of controllably organizing anisotropic nanomaterials into nanostructures with tailored properties, Wang and coworkers [210] programmed anisotropic gold nanorod (AuNR) helix superstructure with tailored chirality. As shown in Fig. 9C, a structure with the longest helix containing 9 AuNRs was obtained by designing the DNA capture strands on both sides of the 2D DNA origami template, arranging them in an “X” pattern, and performing AuNR assembling. The strong chiral–optical activity generated by the AuNR helix manifests potential for various applications, such as chiral fluids, chiral signal amplification, and fluorescence chiral spectroscopy.

The nanostructure programming of DNA origami can also establish 2D networks. Seeman and colleagues [211] constructed DNA origami 2D arrays using cross-shaped origami sheets (Fig. 9D). The construction of DNA origami 2D arrays crucially requires cross-shaped origami sheets whose DNA helices propagate in 2 independent directions. The buildup of curvature in either of the 2 dimensions was prevented by such a design as crystals developed. DNA origami is more likely to form tubular structures if the curvature is not minimized or eliminated [212]. DNA origami technology allows the design and fabrication of discrete 2D DNA shapes with precise positioning of reactive groups [204,213]. Gang and colleagues [214] used a DNA origami octahedron as the basic framework for assembling designed nanoparticle clusters. By placing nanoparticles of different sizes on the same centrosymmetric framework, their optical response, i.e., chiral optical activity, could be fully controlled [106,215,216]. Furthermore, using the octahedral framework as a programmable linker, 0D particles, 1D lines, and square 2D nanoparticle arrays may be readily constructed and produced (Fig. 9E). Moreover, to form nanolattices of flat or curved tubes, Ke and coworkers reported a method to fabricate hexagonal sheets based on DNA origami [217]. To regulate the mechanical characteristics of individual DNA origami sheets as well as the connections between individual sheets, their method blended rational design with input from computer modeling. In designing and preparing nanoscale materials, finding strategies that can self-assemble into arbitrarily designed structures is crucial. Gang and colleagues [218] utilized a set of anisotropic nanoparticle modules that can be selectively combined to create 2D periodic arbitrary shapes (Fig. 9F). Self-assembling DNA components and nanoparticles in a customizable module, leveraging DNA programmability combined with functional nanoparticles, could generate 2D patterns of various designs. This approach opened up enormous possibilities for the design of nanoscale functional materials and devices.

Self-assembling nanoscale materials into 3D structures with precise shapes and dimensions is important in fields such as nanophotonics, metamaterials, and biotechnology [219,220]. Liedl and colleagues [221] used stiff DNA origami scaffolds to build size-controllable hierarchical nanoclusters with a planet–satellite structure out of metal nanoparticles, quantum dots, and organic dyes (Fig. 9G). This technique may be used to create efficient energy funnels, complicated plasmonic structures, and porous nanoengineered catalytic scaffolds. The generation of custom 3D lattices directly using the same set of particles, as well known, is a major challenge in nanotechnology. Gang and colleagues [222] obtained several ordered 3D lattice base structures using functional AuNPs and DNA origami frameworks (DOFs) (Fig. 9H). Using these 3D lattice infrastructures, ordered lattice structures with customizable parameters and elements could be easily artificially additively manufactured. In addition, Liedl and colleagues [223] created a 3D crystal structure made entirely of DNA, allowing for the molecular design of materials and the arrangement of guest particles in a predetermined lattice. Such is a 3D rhomboid lattice with an open structure based on DNA origami and AuNPs, which can be precisely assembled in the lattice and further lays the application potential for metamaterials and structural biology.

Diatoms are particularly good at producing delicate and complex nanostructures as frustule, the main component of which is hydrated silica (SiO_2_·nH_2_O) [224]. The structural symmetry and complexity of diatoms at the nanoscale are difficult to prepare artificially, and their exquisite hierarchical pore structure makes them promising to be used as optical microfilters, microlenses, and other optical components [225]. Recently, Fan and colleagues [226] prepared several siliconized nanostructures based on DNA origami silicification (DOS) technology with patterns resembling the cell wall units of diatoms (Fig. 9I). Compared with pure DNA origami, Young’s modulus of the DOS framework is increased by nearly 10 times, which is enough to support the application of metal nanoparticles in nanoelectronic and nanophotonic devices. Furthermore, Tian and colleagues [227] successfully fabricated DNA origami single crystals with Wulff shape and high yield using a DOF with programmable geometry and binding behavior and then induced an ultrathin silica layer on the edge of the single crystal. Such precise growth leads to a mechanically reinforced silica–DNA hybrid structure (Fig. 9J). Such artificial diatom production procedures would substantially expand the toolset and prospective uses of mesoporous inorganic materials.

DNA origami technology has developed rapidly in the last 10 years, allowing us to customize any shape of lattice at the nanoscale. However, DNA origami technology still faces many challenges, such as high cost, low scale-up, poor stability, and defects. Therefore, we recommend that in the future, DNA origami technology can be further improved by solving the above shortcomings to further explore the application in biosensing, drug delivery, targeted therapy, nano-optics, and other aspects.

### Microscale additive manufacturing

Structural features of natural biomaterials include a hierarchical structure at the microscale and controllable spatial distribution and orientation [9,10,80,228]. The utilization of microscopic additive manufacturing technology to simulate these special structural features may open up a new avenue for manufacturing environmentally friendly biomimetic materials with properties that match or even exceed those of natural materials [41].

**Fig. 10.**
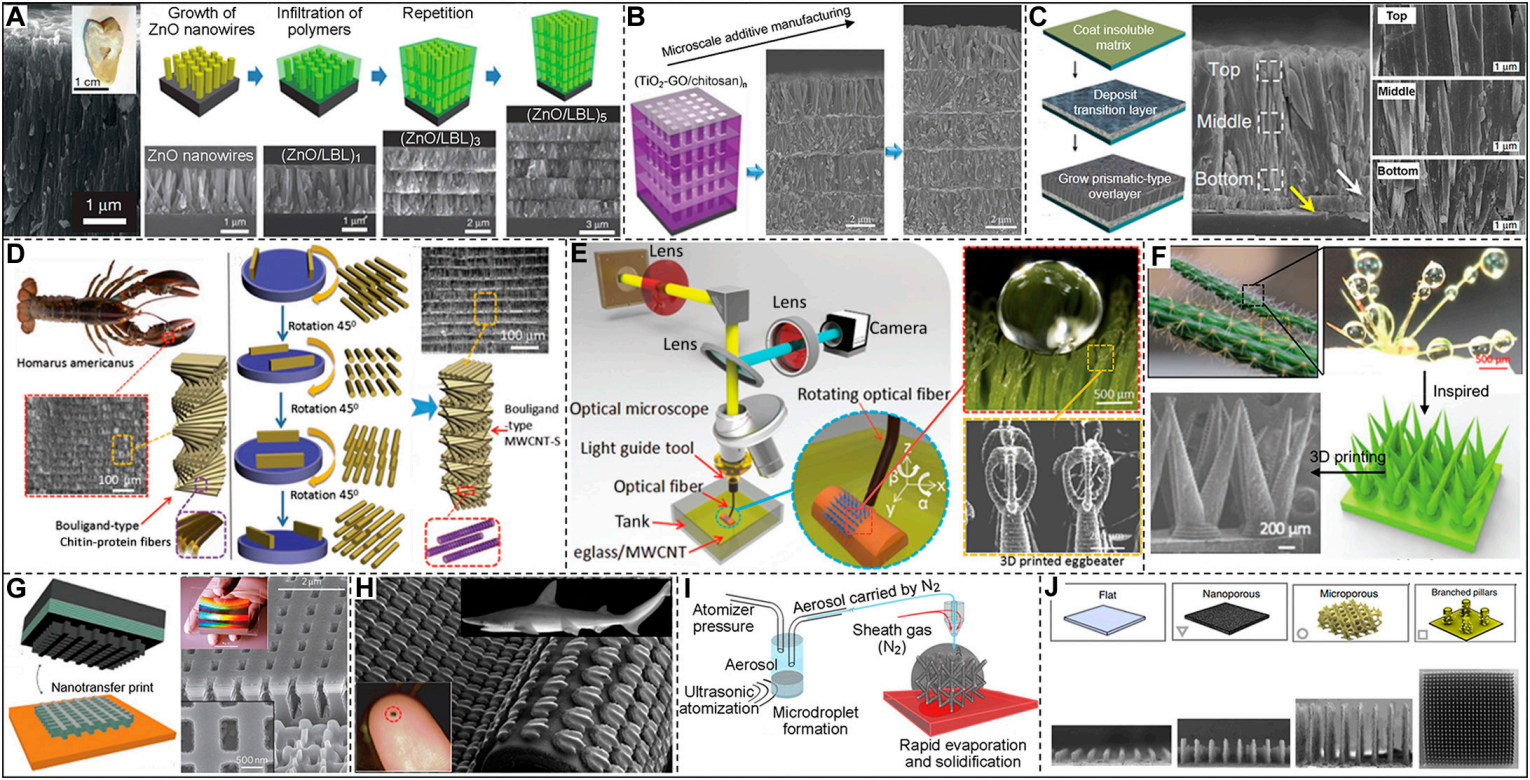
Bioinspired microscale additive manufacturing strategies. (A) The microstructures of natural tooth enamel and abiotic tooth enamel are prepared by the layer-by-layer method. Reproduced with permission [229]. Copyright 2017, Springer Nature. (B) Microscale additive manufacturing of enamel-like composite materials. Reproduced with permission [54]. Copyright 2019, Wiley VCH. (C) Artificial prismatic CaCO_3_ films using a biomimetic mineralization approach. Reproduced with permission [232]. Copyright 2017, Springer Nature. (D) Electric field-assisted 3D printing of Bouligand-type composite structures revealed by lobster chelate microstructure. Reproduced with permission [233]. Copyright 2017, Wiley VCH. (E) 3D printing eggbeater-like structure inspired by the leaves. Reproduced with permission [234]. Copyright 2017, Wiley VCH. (F) A 3D-printed water collection structure inspired by cactus spines. Reproduced with permission [235]. Copyright 2019, Wiley VCH. (G) Structural color thin films prepared by nanotransfer printing. Reproduced with permission [236]. Copyright 2011, Springer Nature. (H) 3D-printed artificial shark skin. Reproduced with permission [238]. Copyright 2014, The Company of Biologists Ltd. (I) Directly 3D printing nanoparticle dispersions in 3D space using aerosol jetting. Reproduced with permission [241]. Copyright 2017, American Association for the Advancement of Science. (J) Large-scale micropillar electrode libraries fabricated by an aerosol jet printing method. Reproduced with permission [242]. Copyright 2022, Springer Nature.

Natural organisms can evolve biocomposites with properties far superior to manufactured materials, and the prominent one is undoubtedly tooth enamel. Kotov’s research team studied the microstructure of natural tooth enamel and used the layer-by-layer method to alternately hydrothermally grow ZnO nanowires on the surface of polyallylamine (PAAm) and polyacrylic acid (PAA) and prepared abiotic enamel materials of mechanical properties comparable to natural enamel (Fig. 10A) [229]. During static nanoindentation testing, they discovered the material’s various benefits, including high strength, good damping, and low density. Further, Wei et al. [54] also used this microscopic additive manufacturing method of layer-by-layer stacking to prepare an enamel-like structure (TiO_2_-GO/chitosan)*_n_* with controllable thickness at the microscale (Fig. 10B). Researchers look forward to such anti-vibration materials and their preparation methods to promote the development of aerospace and other fields. In fact, in mollusk shells, in addition to the presence of nacre, there is also a structure of prismatic layers [230,231]. However, the artificial growth of biomimetic mesoscopic structures with spatial prismatic nacre heterogeneity remains an elusive goal. Cölfen and coworkers [232] successfully prepared prismatic CaCO_3_ films using a biomimetic mineralization approach, including a 3-step pathway: (a) coating of crystalline polymer substrate, (b) deposition of the amorphous transition layer, and (c) mineralizing the prismatic layer (Fig. 10C). The synthesized prismatic CaCO_3_ films exhibit hardness and Young’s modulus comparable to their biological counterparts.

External field-assisted 3D printing can be exploited to produce composites with controllable structural orientation or locally modified surfaces within the selected regions. Chen and colleagues [233] proposed to achieve enhanced biomimetic structures by using electric field-assisted control of the alignment of conductive multiwalled carbon nanotubes (MWCNTs) during 3D printing (Fig. 10D). The electric field-assisted 3D printing method provides insights into the natural toughening mechanism of Bouligand structures and a practical technique for producing synthetic menisci with improved mechanical properties. In addition, Chen and colleagues [234] fabricated an eggbeater-like structure through a surface immersion cumulative 3D printing process inspired by the leaves of *Salvinia molesta* (Fig. 10E). Hydrophilic materials, as demonstrated, can also behave macroscopically as hydrophobic if the surface has the appropriate microstructural features. This 3D-printed eggbeater structure can be applied in water droplet manipulation, 3D cell culture, microreactor, oil–water separation, etc. Chen and colleagues [235] also 3D-printed spines with bioinspired characteristics and special microscopic spatial distribution through the same 3D printing method inspired by the cactus’s surface (Fig. 10F). This study opens prospects for designing next-generation structural materials for efficient energy-free water harvesting.

Developing structurally ordered 2D materials is crucial to the membrane and smart sensing materials. Therefore, 3D printing to prepare microscopically ordered array structures is an important research aspect of bioinspired additive manufacturing. Inspired by the structural color of periodic nanostructures in nature, Rogers and colleagues [236] fabricated negative-refractive-index metamaterials with microscopic 3D structures through a transfer printing process (Fig. 10G). In the near-infrared spectral region, the structure has a refractive index that is obviously negative. The shark is protected and has less frictional fluid resistance due to its skin’s flaky scales and distinctive outer shape [237]. Lauder and colleagues [238] incorporated thousands of hard synthetic shark denticles in a planned linear pattern on a flexible membrane using 3D printing (Fig. 10H). Compared to models lacking the small denticle array, the artificial shark skin created through 3D printing adapted to faster swimming and used less energy to move. In addition, Sun and colleagues [239] used 2-beam interference lithography in conjunction with angle-multiplexed exposure and scanning to print large-area complex structures with cascaded resolution and 3D surface profiles. The developed biomimetic layered structures with different 3D textured surfaces displayed a height accuracy of 0.9 μm to 40 nm and several natural phenomena: superhydrophobicity, iridescence, reflectivity directionality, and polarization of different colors. Furthermore, for the first time, Fabrizio and colleagues [240] utilized surface nanostructures to redesign and fabricate new high-sensitivity sensors. They showed how to use superhydrophobic surfaces to localize molecules in highly dilute solutions at specific locations. Microscopic 3D layered materials are very important for applications of various emerging technologies but are limited by the printing accuracy, because directly conducting a microscale 3D printing is still a challenge. Panat and colleagues [241] provided a method for directly printing nanoparticle dispersions in 3D space utilizing aerosol jetting to create sophisticated 3D microengineered materials such as microlattices (Fig. 10I). The 3D-printed material has almost completely dense truss cells with a minimum diameter of about 20 μm, without using any template or support material. This method may produce layered materials for tissue engineering, lightweight or multifunctional materials, microfluidics, and micro-optoelectronics quickly and mass produced. Recently, Zhang and colleagues [242] also utilized the aerosol jet printing method to efficiently and reproducibly fabricate large-scale micropillar electrode libraries made of indium tin oxide (ITO) nanoparticles (Fig. 10J). In a single printing phase, this printing technique may produce adjustable layered structures spanning 5 orders of magnitude in length scale, which is currently unattainable by other fabrication methods. By employing this method, we have improved our present comprehension of the link between electrode structure and activity and made a milestone in preparing a new generation of semi-artificial photosynthetic systems.

### Micro-macroscale additive manufacturing

The multiscale structure of natural materials endows them with outstanding mechanical properties and functionality. For example, the structure of shells extends from the microscopic orderly arrangement to the macroscopic size, and its performance tremendously exceeds that of the pure-phase minerals composing its structure. Many influential works are accomplished using various bioinspired additive manufacturing methods, such as photocuring, 3D printing, self-assembling, shear flow-induced alignment, layer-by-layer, and scrape coating [192,193,198,243–249]. Here are some typical examples to illustrate the importance of micro-macroscale additive manufacturing to industry and life.

**Fig. 11.**
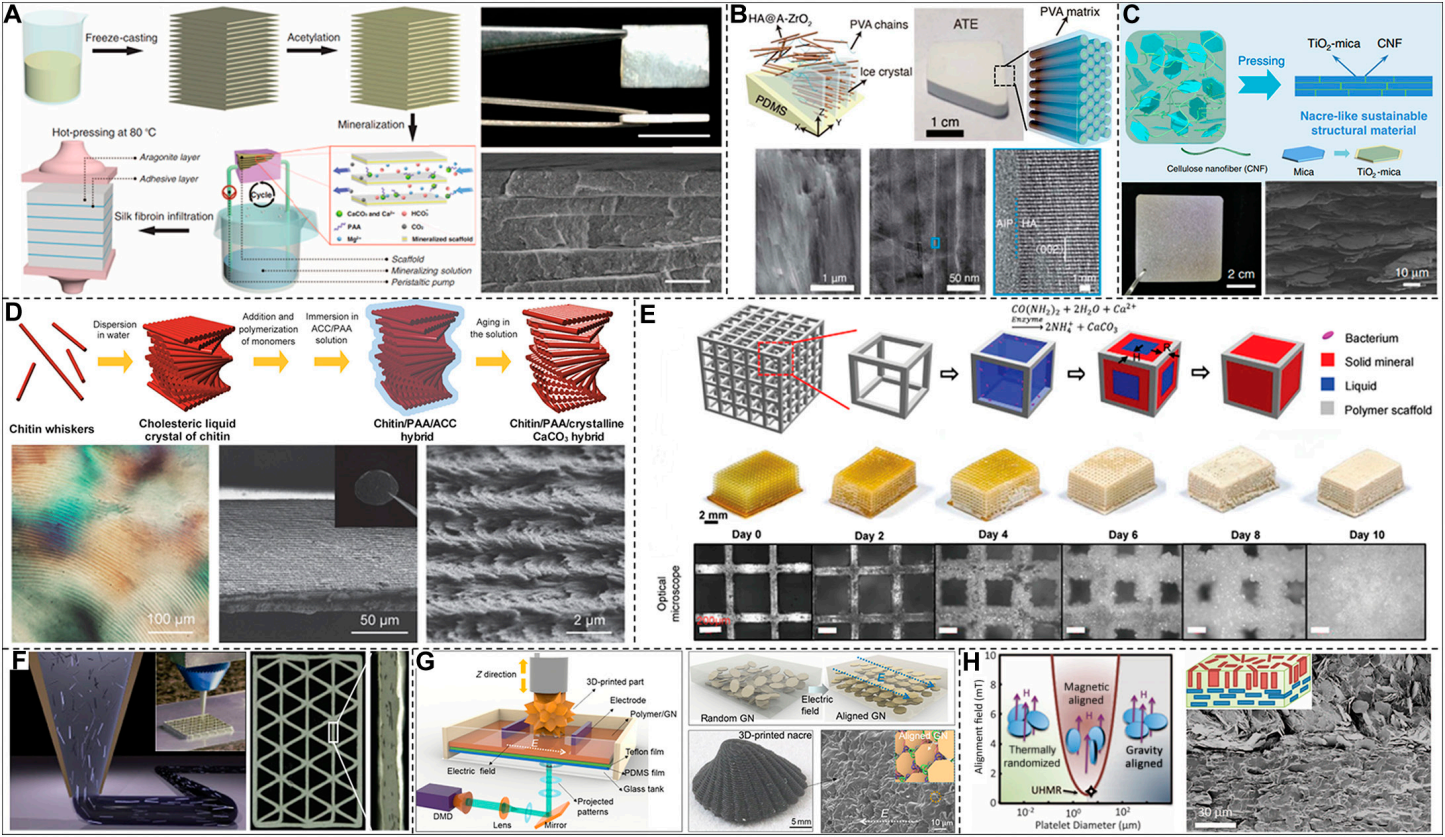
Biomimetic microscale additive manufacturing macroscale architectures. (A) Preparation of synthetic nacre by continuous in situ mineralization assisted by freeze casting. Reproduced with permission [250]. Copyright 2016, American Association for the Advancement of Science. (B) Enamel biomineralization inspired preparation of freeze cast-assisted enamel analog. Reproduced with permission [251]. Copyright 2022, American Association for the Advancement of Science. (C) Self-assembled nacre-like sustainable structural materials. Reproduced with permission [252]. Copyright 2020, Springer Nature. (D) Mineralizing cholesteric liquid crystals of chitin to produce a chitin/CaCO_3_ hybrid. Reproduced with permission [260]. Copyright 2015, Wiley VCH. (E) Mineralized growth of 3D-printed living materials. Reproduced with permission [261]. Copyright 2021, Wiley VCH. (F) Shear-assisted direct ink writing of honeycomb structures. Reproduced with permission [262]. Copyright 2014, Wiley VCH. (G) Seashell-inspired hierarchical structure by electric field-induced 3D printing technology. Reproduced with permission [265]. Copyright 2019, American Association for the Advancement of Science. (H) Magnetic field-assisted preparation of reinforced, wear-resistant, shape memory composites. Reproduced with permission [267]. Copyright 2012, American Association for the Advancement of Science.

Inspired by the mineralization of mollusks in prefabricated layered organic matrices to construct their nacres, Yu and colleagues developed a continuous assembly–mineralization process to prepare synthetic nacres (Fig. 11A) [250]. Synthetic nacres prepared by this mesoscale approach can simultaneously control their nanostructure and microstructure. Briefly, they prepared *β*-chitin with a hierarchical structure through a freeze-induced assembling process, subsequently proceeded in situ mineralization in a solution containing PAA and Mg^2+^, and finally densified through silk fibroin infiltration and hot pressing. Compared with natural nacre, which takes months or even years to grow, this artificially batch-synthesized nacre, which takes only 2 weeks, has almost the same composition as natural nacre, displaying an equivalent mechanical property to natural nacre. Guo and colleagues [251], inspired by the biomineralization of enamel, recently created an artificial enamel with a fundamental hierarchical structure at several sizes by building amorphous intercrystalline phase-coated HAP nanowires with polyvinyl alcohol (PVA) (Fig. 11B). They self-assembled macroscopic artificial tooth enamel (ATE) with parallel-aligned nanowires by bidirectionally freezing a dispersion of amorphous ZrO_2_-coated HAP nanowires in PVA. The researchers further revealed that Young’s modulus and hardness of ATE through nanoindentation reached 105.6 ± 12.1 GPa and 5.9 ± 0.6 GPa, respectively, surpassing natural tooth enamel characteristics. This macro-artificial enamel of high stiffness, hardness, strength, viscoelasticity, and toughness, constructed through a multiscale assembling pathway, manifests the artificial inorganic structures in atomic, nano-, and micro-scales similar to the natural biomineralized materials. In addition, Yu’s team created environmentally friendly building materials with superior mechanical and thermal properties to address the challenge of petroleum-based plastics, which are threatening the environment and human health [252]. As shown in Fig. 11C, this plastic alternative, inspired by the multiscale structure of nacre, was self-assembled from entirely natural basic ingredients (cellulose nanofibers and mica microplatelets) [244]. This material has excellent thermal stability, high strength (281 MPa), high toughness (11.5 MPa m^1/2^), high stiffness (20 GPa), and low thermal expansion coefficient (7 × 10^−6^ K^−1^).

Many organisms orderly assemble biopolymers and inorganic substances into composite structures with excellent physical properties through biomineralization [253–258]. For example, the exoskeletons of crustaceans have high mechanical properties and a helically ordered hierarchical structure composed of CaCO_3_, chitin, and proteins [164,259]. Kato and colleagues [260] used liquid crystal chitin whiskers as a helically ordered template to mineralize a chitin/CaCO_3_ mixture with a helical structure at room temperature (Fig. 11D) and prepared a composite material of structure and composition similar to that of the crustacean exoskeleton. We always find new ideas for creating synthetic materials in biomaterials. However, figuring out how to use living materials to develop substances with predesigned microstructures in engineered systems is still difficult. Here, Wang and colleagues [261] described an effort to create biomimetic mineralized composites with ordered microstructures by utilizing living bacteria and 3D-printed materials (Fig. 11E). This biomineralization technique produced composites with high specific strength and fracture toughness that are on par with natural composites and excellent energy absorption capabilities compared to natural and traditionally artificial composites. This 3D printing method, inspired by the biomineralization process, provides great potential for preparing building materials with ordered microstructures and excellent macroscopic properties.

In addition, macroscopically high-performance biomimetic materials with ordered microstructures can also be easily prepared using external field-assisted 3D printing technology. The honeycomb composite material can not only withstand much of its own weight and wind force but also effectively transport nutrients over long distances to maintain growth [262]. Lewis and colleagues [263] developed a shear-assisted 3D printing process to mimic this lightweight and high-strength structure to obtain polymer resins with well-ordered SiC whiskers and carbon fibers (Fig. 11F). Due to the induction of shear stress during 3D printing, the reinforced walls of the printed honeycomb structure were completed through force alignment. Due to their enormous practical utility, wearable monitoring sensors have garnered growing interest [263,264]. The majority of piezoresistive sensors now being developed, however, are flexible yet unable to shield the human body. To solve this problem, Chen and colleagues [265] used electric field-induced 3D printing technology to construct a seashell-inspired hierarchical structure exploiting aligned graphene nanosheets in a photocurable resin and prepared a smart helmet with a seashell structure (Fig. 11G). The microscale to macroscale assembling of this technology solves the difficulty appearing in traditional processes, in which only simple shell-like films can be fabricated. The smart helmet prepared by electric field-assisted 3D printing has both protective performance and self-sensing ability, prominently enlightening the construction of multifunctional equipment. Magnetic fields have been frequently used in industrial processes due to their versatility in managing the alignment of fillers in polymer resins [266]. For example, Studart and colleagues [267] applied micrometer-sized reinforcing particles coated with superparamagnetic nanoparticles of a minimal concentration (0.01 to 1 vol%) to produce locally reinforced, wear-resistant, shape memory composites (Fig. 11H).

### Macroscale additive manufacturing

Biomimicry is transforming modern materials science and technology by investigating the principles of design found in nature.

**Fig. 12.**
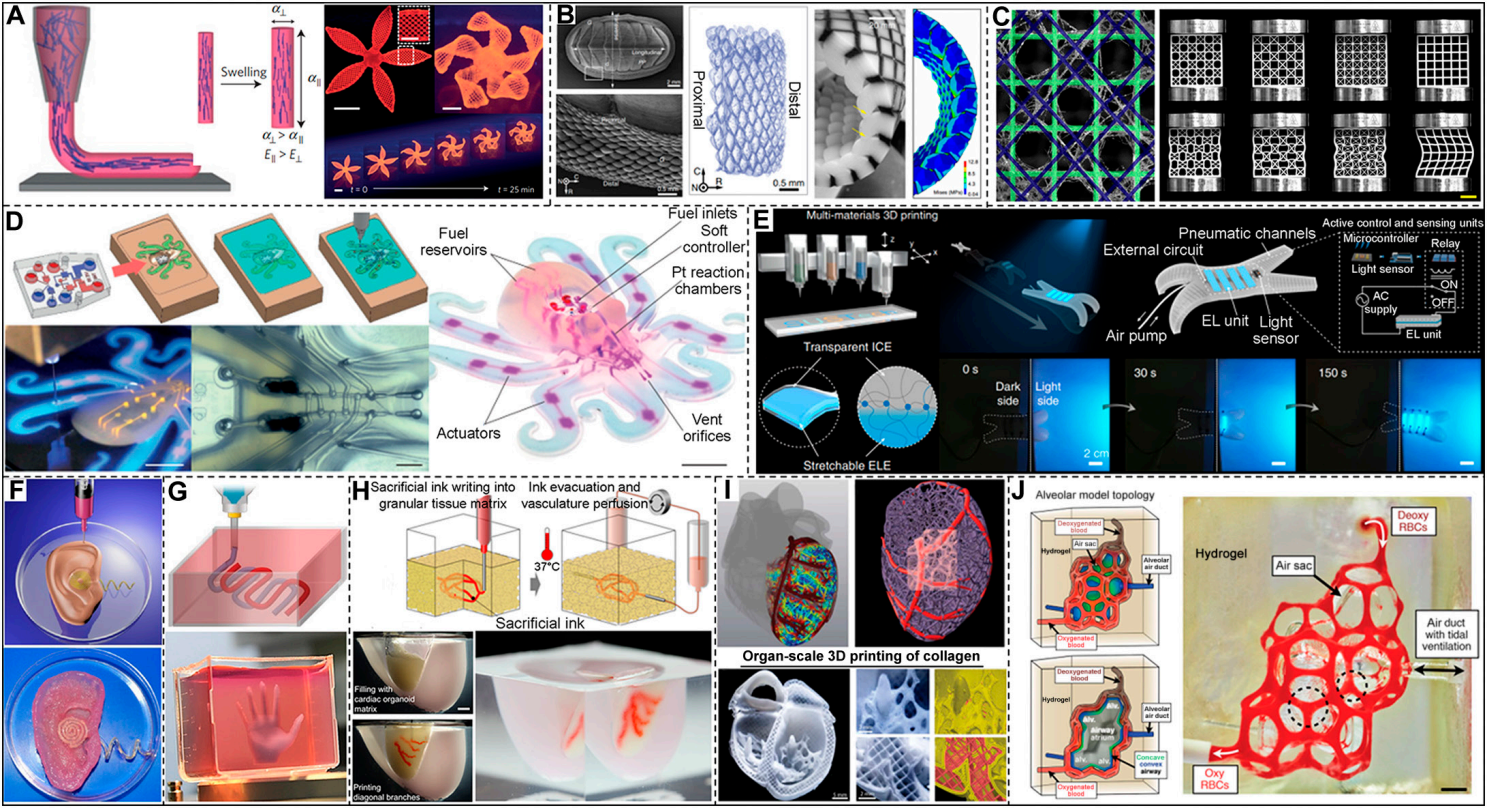
Macroscale additive manufacturing of bioinspired structures and materials. (A) 4D printing of biomimetic hydrogel composite. Reproduced with permission [274]. Copyright 2016, Springer Nature. (B) Multimaterial 3D printing of a synthetic flexible scale armor. Reproduced with permission [275]. Copyright 2019, Springer Nature. (C) Deep-sea sponge-inspired 3D-printed specimens of different lattice geometries. Reproduced with permission [278]. Copyright 2020, Springer Nature. (D) Full 3D printing of flexible robotic octopus. Reproduced with permission [286]. Copyright 2016, Springer Nature. (E) Chameleon-inspired 3D printing of soft robots integrated with flexible light-emitting and light-sensitive units. Reproduced with permission [289]. Copyright 2022, Springer Nature. (F) 3D-printed artificial ear. Reproduced with permission [291]. Copyright 2013, American Chemical Society. (G) Personalized hydrogel is used for 3D printing various types of tissues. Reproduced with permission [292]. Copyright 2019, Wiley VCH. (H) The embedded 3D bioprinting method created a heart tissue fragment. Reproduced with permission [293]. Copyright 2019, American Association for the Advancement of Science. (I) 3D bioprinting of collagen to design human heart components. Reproduced with permission [294]. Copyright 2019, American Association for the Advancement of Science. (J) 3D-printed artificial alveolus. Reproduced with permission [297]. Copyright 2019, American Association for the Advancement of Science.

Buehler and colleagues [268] used computer modeling and microscale 3D printing to investigate the mechanical behavior of elastic webs under various loading circumstances, inspired by the remarkable mechanical properties of spider webs. The findings demonstrate that uniform distribution is better for confined loads, whereas stronger radial threads and weaker helical threads are preferable for spread loads. Complex shapes and the functions of the material are tightly associated with natural selection and evolutionary optimization. By comparing the mechanical stabilization of 3D-printed artificial shells with natural shells, Tiwary et al. [269] demonstrated that complex shapes could withstand almost twice the load than simple shapes. Through finite element analysis (FEA), an important causal relationship was illustrated between the natural shape of the shell and the respective mechanical stability. Plants undergo hydration-induced morphological changes due to variances in local swelling caused by the orientation of stiff cellulose fibers within the plant cell wall [270–273]. Lewis and colleagues [274] were motivated by this, and they created a bioinspired hydrogel that can be used for 4D printing to create a programmable bilayer structure that is patterned in both space and time (Fig. 12A). The rigid cellulose nanofibers in this hydrogel were enclosed in a supple acrylamide matrix. Thus, the local control of the orientation of internal cellulose fibers enables its biomimetic 4D printing, displaying anisotropy of elasticity and swelling. The design of such biocompatible flexible ink will inspire the development of new approaches for tissue engineering, biomedical devices, soft robotics, etc. Conch shells are one of the hardest tissues in nature, one order of magnitude harder than nacre [97]. But its intersecting lamellar structure is difficult to realize by exploiting synthetic materials. Buehler and colleagues [99] prepared a conch-like macrostructure material by 3D printing. Combining a comprehensive approach of simulation and drop tower testing, the findings suggest that the main mechanism of conch shell shock resistance is the production of deflected paths of fracture, which can be used in the design of future protective equipment such as hoods and body armor. Contemporary artificial armor usually relies on rigid structures for mechanical protection but often lacks sufficient flexibility [275]. Chitons have strong teeth, but moreover, they have also evolved scales for defense [276]. The orderly distribution of scales ensures the flexibility of movement and protects the underlying soft body [277]. Inspired by this, Li and coworkers [275] used multimaterial 3D printing to fabricate a synthetic flexible scale armor (Fig. 12B). By parametric computer modeling, they investigated the practical trade-offs between this armor system’s protection and flexibility while assessing its potential to further the creation of additional useful prototypes. The skeleton system of the deep-sea sponge *Eulectella aspergillum* is a square grid-like structure composed of amorphous hydrated silica with certain toughness and strength [112]. In various lattice geometries of 3D-printed samples using a combination of finite element models and mechanical tests, Bertoldi and colleagues [278] showed that the diagonal reinforcement strategy of the sponge could achieve the highest resistance to buckling for quantitative materials (Fig. 12C). The knowledge obtained from their study of the sponge-skeleton system can be used to realize the geometric optimization of the square lattice to avoid buckling of the global structure, which is important for optimizing materials applied in modern infrastructure.

Conventional robots are usually composed of multiple rigid materials, thus severely limiting their applications [279,280]. Soft robots possess many outstanding properties attributed to the structural softness of their constituent materials. However, the soft robot must remain connected to an external control system and power supply [281–285]. Wood and colleagues [286] report unrestricted manipulation of robots composed only of soft materials to address this issue. Molding and soft lithography created the soft robot’s body and microfluidic logic, as illustrated in Fig. 12D. The pneumatic actuator network, onboard fuel storage tank, and catalytic reaction chamber required for movement were fabricated by multimaterial embedded 3D printing technology [287,288]. This integrated design and quick manufacturing technique allow for the programmed assembly of numerous materials inside this architecture, illuminating entirely soft autonomous robots. In addition, Liu’s team was inspired by the chameleons in nature. Thus, they integrated flexible light-emitting and light-sensing units on soft robots through ink-based 3D printing technology and built an artificial intelligence camouflage system (Fig. 12E) [289]. By using 3D-printed electroluminescent devices in conjunction with soft quadruped robots and sensor modules, the robot can autonomously perceive environmental changes during walking and can quickly respond to camouflage feedback, manifesting the potential to develop next-generation soft robots.

Every organ in the human body is an extremely complex tissue system. Among the countless methods to develop artificial organs, 3D bioprinting offers unparalleled potential [290]. As a proof of concept, McAlpine and colleagues [291] designed a bioinspired ear by 3D printing a cell-seeded hydrogel matrix infused with interwoven conductive polymers composed of silver nanoparticles (Fig. 12F). The auditory induction toward the receiving radio frequency is amplified in the printed ear. In addition, Dvir and colleagues [292] used personalized hydrogels as bioinks to develop and apply advanced 3D printing techniques. The personalized hydrogel is reprogrammed and processed from omentum tissue removed from the patient. As shown in Fig. 12G, as a proof of concept, they printed it into various types of tissues, such as the hand palm. These results demonstrate the method’s potential for engineering personalized tissues and organs. In addition, Lewis and colleagues [293] described a 3D bioprinting technique that uses organoids made from patient-specific induced pluripotent stem cells as building blocks for organs to produce tissues with cell density, microstructure, and function (Fig. 12H). They generated a heart tissue piece with a perfusable microstructure that fused and beat synchronously in 7 days to show the printed organs’ functionality. Furthermore, Feinberg and colleagues [294] suggested a technique for 3D bioprinting collagen that uses free-reversible embedding of suspended hydrogels (FRESH) to construct human heart components at multiple sizes, from capillaries to whole organs. As shown in Fig. 12I, they used cardiomyocytes and collagen to create a left ventricular model. They then further examined the electrophysiological behavior and ventricular contraction events related to heart rate abnormalities. In human organs, the vascular system and other pipeline systems intertwine in a very complex 3D topology, thus fulfilling physiological functions such as material transport and exchange [295,296]. These properties of the vascular system pose a great challenge to scientists. Miller’s team used 3D printing technology associated with stereographic projection lithography to prepare a 3D functional intravascular system with food color additives as biocompatible light absorbers, displaying the intravascular mitral valve function [297]. Therefore, the researchers printed an artificial alveolus, which can exchange gas with the vascular system and thus obtain oxygen like human alveoli during simulated breathing (Fig. 12J). This tiny alveolus has only a coin size, but it is a milestone for artificial organs.

## Conclusions and Outlook

Biological systems have evolved over billions of years, resulting in complex structures and functions that enable them to endure environmental hazards. Bioinspired materials transformed modern engineering and technology, taking inspiration from nature’s design principles and concepts. Most bioinspired materials currently have only one functional property. However, various technologies that symbolize current scientific progress, such as biointegrated electronics, biomimetic robots, high-performance batteries, and biodegradable plastics, require combining at least 2 to 3 materials with basic properties and a broad range of uses. Bioinspired multifunctional composite materials with multiscale structural designs can achieve high performance and energy efficiency. However, realizing nature-inspired design requires new material manufacturing methods for multiple-scale assembly. Nowadays, graded nanocomposites demonstrate a blend of mechanical, electrical, optical, and biological properties in almost all circumstances due to their fabrication using a wide range of techniques. Such a combination of properties is not feasible with traditional materials. These functions represent the main advantages of bioinspired material preparation methods and reflect the principles of material synthesis in living organisms. Unfortunately, the current single approach to bioinspired preparation limits the development and application of functional composites. A combination of bioinspired design and advanced manufacturing techniques will be a prerequisite to overcome this limitation.

**Fig. 13.**
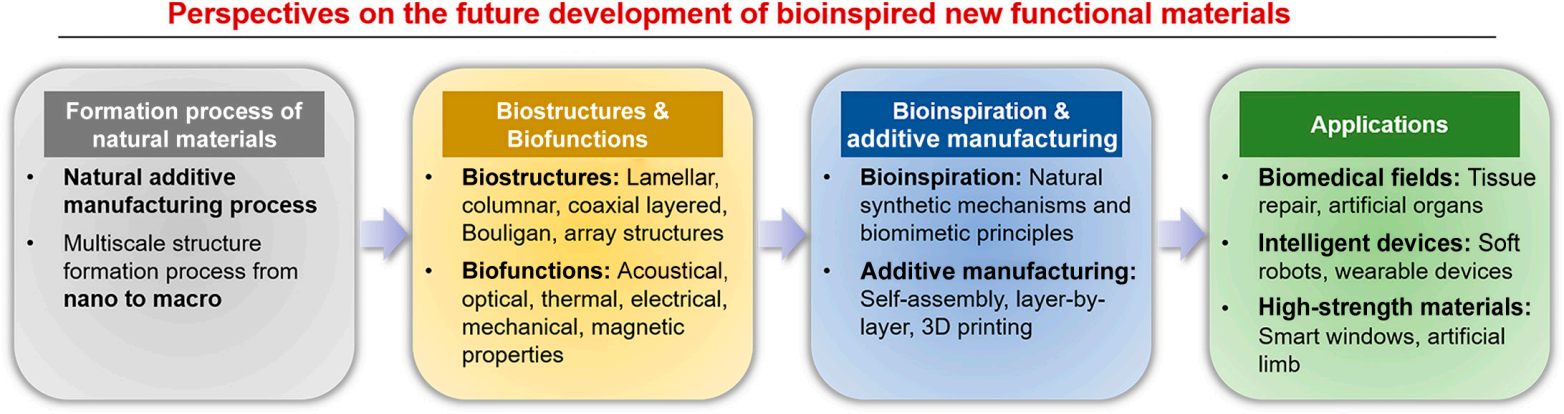
Future directions for additive manufacturing of bioinspired composites.

The most important feature of natural biostructured materials is their macroscopic 3D structure, created by the multiscale assembling of nanostructured units. This bottom-up self-assembling multiscale structure is a natural additive manufacturing process in living organisms, reflecting structural and property optimization at each scale. Materials science and technology face a challenge in designing and preparing cross-scale structural materials with special properties using current technologies to precisely control the internal microstructures. Additive manufacturing technology has the potential to solve this challenge. Additive manufacturing, including self-assembling, 3D printing, layer-by-layer, and scratch coating, has been utilized to fabricate bioinspired composites with outstanding properties. The goal is to employ multiscale additive manufacturing technologies from nano to macro, investigate the development from limited material combinations to the fusion of multifunctional materials, and extend the applications from demonstrations to life. Therefore, we provide insights into new functional materials fabricated by bioinspired additive manufacturing, as shown in Fig. 13.

To achieve this goal, the structure of biological systems needs to be explored to understand the natural additive manufacturing process involved in forming natural materials. The multiscale structural composition from nano to macro needs to be analyzed. Starting from the basic nano-units composing biological structures, various natural biological structures need to be studied to explore the functions of biomaterials in different aspects. The structure–function relationships need to be analyzed to provide theories for the future design and implementation of bioinspired functional materials.

Moreover, the design and realization of bioinspired functional composites need to be extended to cross-scale and multisystems by taking the structure and function of typical biomaterials as studying objects. Additive manufacturing technologies such as self-assembling, layer-by-layer, and 3D printing can be used to develop new multifunctional materials by applying their natural synthesis mechanism and design principles under environmental conditions. By studying biomaterials’ structure formation and function, the preparation of bioinspired composites can be guided. Manufacturing bioinspired composites through additive manufacturing can deepen our understanding of biological design principles.

Finally, the applications of new bioinspired functional materials fabricated by additive manufacturing need to be investigated in biomedicine, artificial intelligent devices, and high-strength materials based on the structure–function relationship. The evaluation system of new bioinspired functional materials fabricated by additive manufacturing should be established regarding sound, light, heat, electricity, mechanics, and magnetism to provide criteria for evaluating new materials.

As research progresses, it is expected that multiscale additive manufacturing technologies and bioinspired design principles will become more integrated, paving the way for the design and manufacturing of advanced functional composites.
